# Insights into volcanic hazards and plume chemistry from multi-parameter observations: the eruptions of Fimmvörðuháls and Eyjafjallajökull (2010) and Holuhraun (2014–2015)

**DOI:** 10.1007/s11069-023-06114-7

**Published:** 2023-08-19

**Authors:** Amy Donovan, Melissa Pfeffer, Talfan Barnie, Georgina Sawyer, Tjarda Roberts, Baldur Bergsson, Evgenia Ilyinskaya, Nial Peters, Iris Buisman, Arní Snorrason, Vitchko Tsanev, Clive Oppenheimer

**Affiliations:** 1 University of Cambridge, Cambridge, UK; 2 Icelandic Met Office/Veðurstofa Íslands, Bústaðavegi 7-9, 105 Reykjavík, Iceland; 3 Department of Electronic and Electrical Engineering, Faculty of Engineering, University College London, Gower Street, London, UK; 4 Laboratoire de Physique et de Chimie de l’Environnement et de l’Espace, CNRS, Université d’Orléans, Orléans, France; 5 Laboratoire de Météorologie Dynamique, IPSL, CNRS, Ecole Normale Supérieure, Sorbonne Université, PSL Research University, Paris, France; 6 School of Earth and Environment, University of Leeds, Leeds, UK; 7 Department of Geography, University of Cambridge, Downing Place, Cambridge, UK; 8 Department of Earth Sciences, University of Cambridge, Downing Place, Cambridge, UK

**Keywords:** Volcanic gas hazard, Volcanic gas monitoring, Iceland, Multi-parameter monitoring, Volcanic plumes

## Abstract

The eruptions of Eyjafjallajökull volcano in 2010 (including its initial effusive phase at Fimmvörðuháls and its later explosive phase from the central volcano) and Bárðarbunga volcano in 2014–2015 (at Holuhraun) were widely reported. Here, we report on complementary, interdisciplinary observations made of the eruptive gases and lavas that shed light on the processes and atmospheric impacts of the eruptions, and afford an intercomparison of contrasting eruptive styles and hazards. We find that (i) consistent with other authors, there are substantial differences in the gas composition between the eruptions; namely that the deeper stored Eyjafjallajökull magmas led to greater enrichment in Cl relative to S; (ii) lava field SO_2_ degassing was measured to be 5–20% of the total emissions during Holuhraun, and the lava emissions were enriched in Cl at both fissure eruptions—particularly Fimmvörðuháls; and (iii) BrO is produced in Icelandic plumes in spite of the low UV levels.

**Supplementary Information:**

The online version contains supplementary material available at 10.1007/s11069-023-06114-7.

## Introduction

Icelandic volcanoes present a range of hazards, and have become widely known in particular for ash and gas emission, and jökulhlaups (glacial floods; Barsotti et al. 2020; Bird et al. 2009; Guðmundsson et al. 2008). Historical eruptions have been associated with large emissions of sulphur dioxide and concomitant climatic forcing (Oppenheimer et al. 2018; Schmidt et al. 2011; Thordarson and Self 1993). In 2010 and 2011, ash clouds from subglacial eruptions disrupted aviation (Donovan and Oppenheimer 2010; Icelandic Meteorological Office 2012; Weber et al. 2012), and in 2010 had significant local impacts on agriculture (Bird and Gísladóttir 2018, 2012) which can be related to volcanic halogens such as HF being deposited with ash (Bagnato et al. 2013). The flooding produced by such eruptions has also received attention (Alho et al. 2007; Dugmore et al. 2013; Eliasson et al. 2006; Pagneux et al. 2015).

Risks from Icelandic eruptions received global attention during the “ash crisis” of 2010 (Donovan and Oppenheimer 2010; Icelandic Meteorological Office 2012; Parker 2015), largely because of far-field impacts of volcanic ash on aviation. Substantial investment since 2010 has enabled increased monitoring of volcanic eruptions (Sigmundsson et al. 2013), including, increasingly, volcanic gases (Pfeffer et al. 2018). Historical research concerning the 1783–4 eruptions of Lakagígar has also demonstrated repeatedly that volcanic gas impacts on Iceland and on the climate system can be very significant (Schmidt et al. 2011; Thordarson and Self 1993). The 1783–4 eruptions are associated with summer cooling and winter warming in the Northern Hemisphere—and with large-scale local impacts in Iceland (Oppenheimer 2011): the Moðuharðindin (“Mist hardships”) caused the death of over half Iceland’s livestock from fluorosis, leading to a major famine (Gestsdóttir et al. 2006; Walser et al. 2020). There is thus historical evidence that Icelandic eruptions can produce very substantial SO_2_ and halogen emissions that have devastating local impacts. Understanding gas emissions and their variation in such systems is therefore an important aspect of hazard assessment and scenario construction.

In this paper we compare and contrast multidisciplinary datasets from the eruptions at Fimmvörðuháls and Holuhraun. We look in particular at the emissions of halogen species in both cases, and at the hazards presented in the near-field.

### Recent Icelandic eruptions

**Fig. 1:**
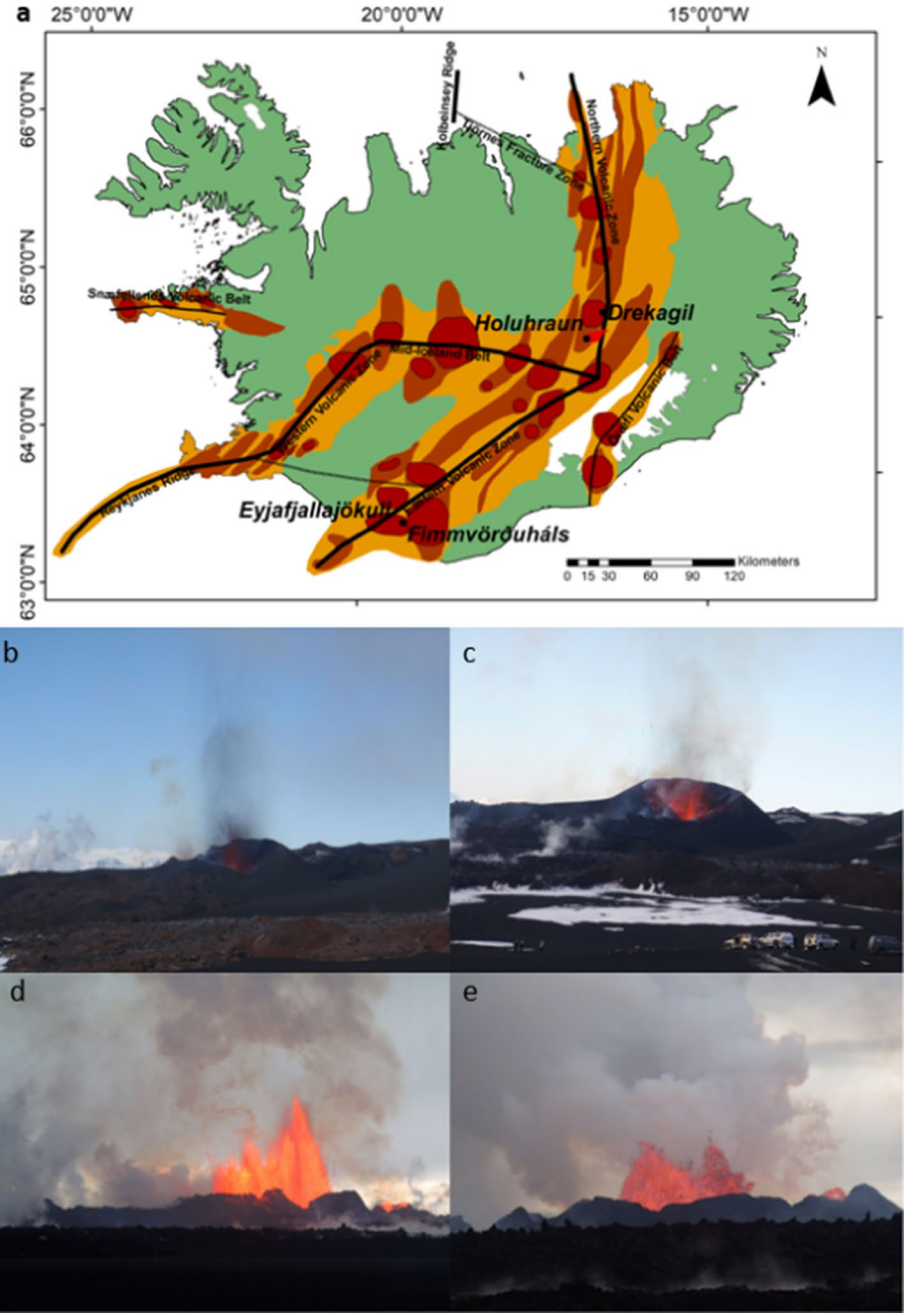
Map of Iceland (Upper), showing the main volcanic zones in orange, fissure systems in dark brown and volcanic centres in dark red. The Holuhraun flow field is show in bright red, and the main tectonic features in black lines. Key locations are marked. Lower panel: photographs of fire fountain activity at **b**, **c** Fimmvörðuháls and **d**, **e** Holuhraun

The 2010 eruption of Eyjafjallajökull was preceded by almost 20 years of intermittent unrest (Sturkell et al. 2003). In 1994 and 1999, magma intrusion was detected under the southern slopes of the volcano (Pedersen et al. 2007; Pedersen and Sigmundsson 2004; Sturkell et al. 2003), and shallow seismicity was detected in 2009—2010. On 20 March 2010 at 23.25 UT (local Icelandic time + 00:00) the initial phase of the eruption, at Fimmvörðuháls (Fig. 1), commenced. It was an effusive, lava-rich and ash-poor eruption centred along a 1 km NE-SW fissure on the north-eastern flank of the volcano at ~ 1000 m altitude. Lava fountaining and lava flows were observed, with lava fountains reaching around 100 m in height and producing a low-altitude ash-poor plume (Edwards et al. 2012; Ilyinskaya et al. 2012). This fissure eruption ceased on 12 April, but early on 14 April, an explosive ash-rich summit eruption began. Initially subglacial, it triggered a significant jökulhlaup (glacial melt-water flood) but by daybreak on 14 April a dense ash plume was observed, reaching 8–10 km a.s.l (Arason et al. 2011). Four days of sustained ash and tephra production followed, disrupting air traffic across the Atlantic and closing airspace over much of northern Europe (Guðmundsson et al. 2012; Sigmundsson et al. 2010). The eruption continued until 23 June, with periodic disruption of airspace particularly during the first month.

Other authors (Moune et al. 2012; Sigmarsson et al. 2011) have demonstrated that the lava from the Fimmvörðuháls eruption had a deep source (16–18 km), intermediate in composition between Surtsey and Katla lavas, and that the later summit eruption was triggered by mixing of this melt with a more evolved melt under the summit. Prior to the flank eruption, the magma was emplaced into two sills at shallow depths (Keiding and Sigmarsson 2012; Sigmundsson et al. 2010). Additionally, the mixing of the more primitive melt with sills beneath the Fimmvörðuháls area (Sigmundsson et al. 2010) was supported by Moune et al. (2012) based on variations in the trace element geochemistry of the erupted products. They found anomalously high levels of Cl in one melt inclusion, suggesting either a xenocryst or the presence of a Cl-rich brine at depth. The S/Cl ratio of the Fimmvorðuháls basalt plots on a linear trend at the lower end of a range from Veiðivötn in the northeast to Surtsey in the south (Moune et al. 2012), consistent with a mantle plume-dominated rather than depleted MORB-influenced origin (Aiuppa 2009; Halldórsson et al. 2016). Trace element abundances and ratios further showed the likely presence of recycled oceanic crust in the primitive melt (Moune et al. 2012).

The 2014–2015 Holuhraun eruption sourced from Bárðarbunga volcano under Vatnajökull occurred 47 km from the central volcano just north of the icecap on the *sandur* (glacial flood plain; Guðmundsson et al. 2016; Pedersen et al. 2017). It started on 29 August 2014 from a small fissure, followed by a brief hiatus before the main fissure opened on 31 August. The eruption has been very well documented in the literature and; while, it was an order of magnitude smaller than the 1783–4 Skaftar Fires (Laki) eruption, it nonetheless has become regarded as a modern archetype for large basaltic eruptions with high sulphur contents (Gíslason et al. 2015; Ilyinskaya et al. 2017; Pfeffer et al. 2018; Schmidt et al. 2015; Stefánsson et al. 2017). The eruption continued until 27 February 2015, with activity waning through the later months (Pfeffer et al. 2018).

Holuhraun, similarly to the Fimmvörðuháls eruption, had a relatively deep source at 17–18 km (Geiger et al. 2016), with the most primitive melt inclusions indicating equilibration pressures equivalent to around 25 km depth (Hartley et al. 2018). However, the magma was likely stored in the mid-crust (6–7 km) prior to eruption (Halldórsson et al. 2018), which would have allowed the gases to modify from equilibrium with the deep source magma towards equilibrium at this shallower level. Halldórsson et al. (2018) also note that the temporally uniform melt was formed by the mixing and crystallization of multiple primary melts as it moved along the dyke from the central volcano to the eventual eruption site. Melt chemistry also shows that the magma, like other Icelandic magmas, was more oxidised than typical MORB; and therefore, relatively sulphur-rich when the magma reached the surface (Halldórsson et al. 2018; Hartley et al. 2018; Shorttle et al. 2013, 2015).

Melt inclusion studies of samples from Holuhraun suggest that the ratio of S/Cl is much higher than that at Eyjafjallajökull, and consistent with a depleted MORB source (Bali et al. 2018). The Cl in particular showed substantial variation in the melt inclusions, though the most primitive showed a range of 47–71 ppm, which is consistent with values in the literature for MORB (Aiuppa et al. 2009). Chlorine solubility in water-poor melts is high (Webster et al. 1999, 2009), and so Cl can remain in the melt without significant degassing prior to eruption. However, the behaviour of halogens in Icelandic systems remains relatively difficult to constrain, partly because of high variations in the halogen contents of melt inclusions and glasses, and high errors on volcanic gas measurements. This is complicated by the apparent heterogeneity of mantle sources under Iceland (Halldórsson et al. 2016, 2018; Shorttle et al. 2013; Winpenny and Maclennan 2014), including mantle volatile heterogeneity (Hartley et al. 2021; Ranta et al. 2022). Sigmarsson et al. (2020) present data for S, Cl, and F in the residual (post-eruptive) period and show that the halogens were higher in the residual gas relative to sulphur than in the syn-eruptive gas. They suggest that this may relate to an influence of S depletion on the solubility of Cl in the magma, but that this requires further investigation.

Recent eruptions in Iceland have provided the opportunity to measure volcanic gases at high latitude using UV spectroscopy and other methods in this unique tectonic setting (Gíslason et al. 2015; Ilyinskaya et al. 2017; Pfeffer et al. 2018; Schmidt et al. 2015; Stefánsson et al. 2017). While UV-DOAS has been used for three decades now to monitor volcanoes, many of those most widely monitored are located in temperate to tropical regions, benefitting from stronger UV radiation (Bobrowski et al. 2007; Bobrowski and Platt 2007; Kern et al. 2009). Measurements using DOAS have been made at Erebus volcano in Antarctica; these are made at the height of the southern summer (Boichu et al. 2011). Measurements in Alaska have also been made during the northern summer (Kelly et al. 2013; Lopez et al. 2017). Using UV spectroscopy, it has been shown that halogen compounds in volcanic plumes can rapidly oxidise via photochemical cycles to form BrO (and more rarely OClO) (e.g. Bobrowski and Platt 2007; Donovan et al. 2014; Oppenheimer et al. 2006, Gutmann et al., 2018). Satellite remote sensing (Heue et al. 2011; Rix et al. 2012; Schmidt et al. 2015), and ground-based methods (Gíslason et al. 2015; Ilyinskaya et al. 2017; Pfeffer et al. 2018) have identified halogens in Icelandic eruption plumes. However, there are some challenges in using UV spectroscopy in Iceland, particularly in the winter months when the solar UV source is particularly limited and photolysis may be curtailed. Measurements at both of the eruptions discussed in this paper took place close to the equinox when the UV spectroscopic retrievals can have the least scattering due to solar pathlength (in March–April 2010 and September 2014), and at both, BrO was detected (Heue et al. 2011; Hörmann et al. 2013).

BrO is not a primary magmatic product, but is produced through the following autocatalytic reaction cycle within the gas plume, including a photolysis step:

1 $${\text{HBr}} + {\text{OH}} \to {\text{Br}} + {\text{H}}_{{2}} {\text{O}}$$

2 $${\text{Br}} + {\text{O}}_{{3}} \to {\text{BrO}} + {\text{O}}_{{2}}$$

3 $${\text{BrO}} + {\text{HO}}_{{2}} \to {\text{HOBr}} + {\text{O}}_{{2}}$$

4 $${\text{HOBr}} + {\text{HBr}}_{{({\text{aq}})}} \to {\text{Br}}_{{2}} + {\text{H}}_{{2}} {\text{O}}$$

5 $${\text{Br}}_{2} + hv \to 2{\text{Br}}$$

6 $${\text{net}}:{\text{HO}}_{{2}} + {\text{Br}} + {\text{O}}_{{3}} + {\text{HBr}}_{{({\text{aq}})}} + h\nu \to {\text{2BrO}} + {\text{2O}}_{{2}} + {\text{H}}_{{2}} {\text{O}}$$

where *hν* represents the energy absorbed by the reactants (*h* is Planck’s Constant and *ν* is the frequency of the radiation). As part of the reaction cycles, BrO interconverts rapidly between other forms of reactive bromine, including HOBr, Br_2_, BrCl, Br, BrONO_2_, and BrNO_2_. The model of von Glasow (2010) suggests reactive bromine in the young (< 1 h) plume from Etna is primarily in the form of BrNO_2_ due to reaction of Br with NO_2_, although other models that simulate both BrNO_2_ isomers found BrNO_2_ to be much less prevalent (Roberts et al. 2014). Roberts et al. (2009) highlight the role of BrONO_2_ alongside HOBr in propagating reactive bromine formation: once formed Eq. (7), BrONO_2_ may react on aqueous aerosol, forming intermediate product HOBr Eq. (8) that can go on to react with Br^−^ to produce Br_2_ Eq. (4).

7 $${\text{BrO}} + {\text{NO}}_{{2}} \to {\text{BrONO}}_{{2}}$$

8 $${\text{BrONO}}_{{2}} + {\text{H}}_{{2}} {\text{O}}_{{({\text{l}})}} \to {\text{HOBr}} + {\text{HNO}}_{{3}}$$

Recent models and observations (Jourdain et al. 2016; Roberts et al. 2014) suggest that an increase in BrO/SO_2_ in the near-source plume should be followed by a decrease a few hours downwind. The data presented in this paper back up this suggestion.

Halogen behaviour in volcanic systems has mostly been studied at subduction zones. The behaviour of Cl in magmatic systems is known to be highly complex and dependent on a range of intrinsic variables (Zajacz et al., 2012; Alletti et al. 2009; Beermann et al. 2015; Humphreys et al. 2009; Sigmarsson et al. 2020). Partitioning of halogen species between melt and fluids is relatively poorly constrained and again the focus has been on subduction zones and on more evolved magmas. Cadoux et al. (2018), in experiments on arc magmas, found that Br partitioning, like that of Cl, is strongly compositionally controlled and is also affected by temperature (with *D*_Br_^*f*/*m*^ increasing with decreasing temperature, and with increasing SiO_2_ content). The influence of pressure was inconclusive but not consistent with the results of Bureau et al (2010), who found a strong increase in the partition coefficient with decreasing pressure in silicic compositions. As a result of these limitations, their data are not particularly helpful in constraining processes in the conduit or at the surface—particularly in a non-subduction context, since almost all studies have focussed on arc magmas (Balcone-Boissard et al. 2010; Bureau et al. 2000, 2010; Cadoux et al. 2018; Donovan et al. 2014). Cadoux et al (2018) further develop an S–Cl–Br degassing model that suggests that the gas phase will initially be high in S, but as S becomes degassed, will become higher in Cl and Br (Cadoux et al. 2018). We use our data to analyse the different degassing pathways at Eyjafjallajökull and Holuhraun. We supplement the limited data available for volcanic gas emissions from Icelandic volcanoes with new observations, and evaluate their implications for monitoring and management of volcanic hazards from eruptions. In particular, we report (i) observations of BrO/SO_2_ ratios for the emissions from Fimmvörðuháls, Eyjafjallajökull and Holuhraun; (ii) observations of SO_2_ fluxes associated with the eruption of Eyjafjallajökull; and (iii) examine periodicity in these and other gas datasets.

This paper focuses particularly on comparing degassing from the two volcanic systems, and presents the results from each eruption in turn (Fimmvörðuháls and Eyjafjallajökull, both sourced from the Eyjafjallajökull central volcano, and then Holuhraun, sourced from Bárðarbunga central volcano). The discussion then takes forward the comparison between the volcanic systems in relation to wider literature, simulates the chemistry of BrO in the plume, and discusses the outlook for monitoring of Icelandic fissure eruptions.

## Methods

### UV spectroscopy

All UV spectra were collected using an Ocean Optics USB2000 + spectrometer mounted on a tripod, with data acquisition controlled by DOASIS software (Kraus 2006). Stationary observations, with fixed fields of view, were made for measurement of SO_2_ and BrO abundances, and traverses beneath the plume carried out for flux measurements.

**Fig. 2:**
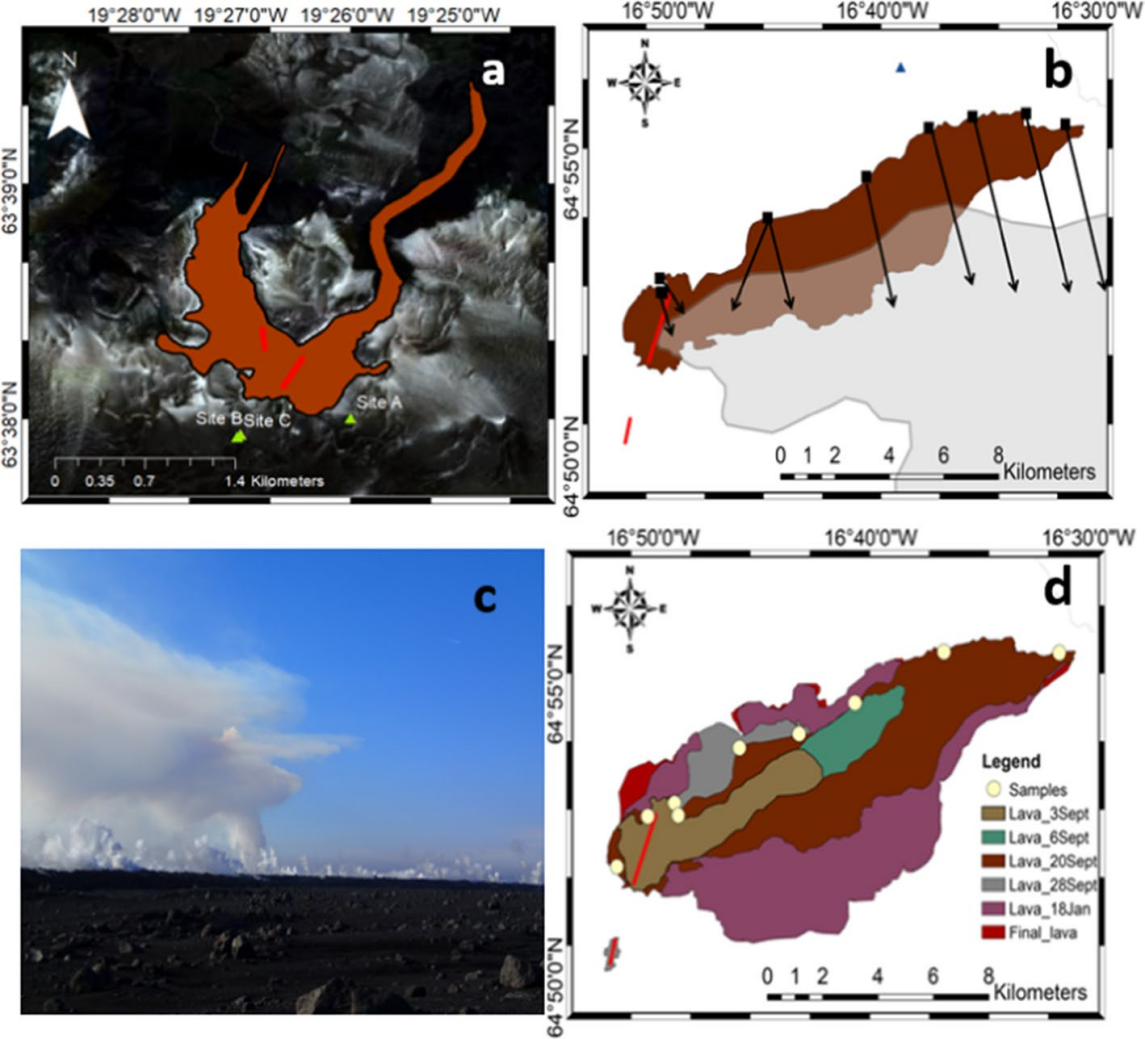
**a** Location of measurements at Fimmvörðuháls—sites A and B were used 28 March, 1 and 4 April. Site C was used on 7th April due to presence of tourists at the other sites, but is almost identical to Site B. Red lines indicate fissures. **b** Locations of stationary measurements made on 20 September 2014 at Holuhraun, with approximate plume location (grey) and lava extent (brown) on that date sketched from satellite imagery; red lines indicate fissures and arrows indicate the direction of view of the spectrometer from fixed positions. Blue triangle is location of **c**—Photograph of the Holuhraun plume on 20 September 2014. Note the low-level cloud coming from the flows, partly due to flow degassing and partly a result of interactions with groundwater. **d** The sample locations are shown in cream. The lava flow field at the time of sampling (20 Sept) is dark brown. Other colours show the evolution of the flow field during the entire eruption: olive—3 Sept; green—6 Sept; grey—28 Sept; purple—early January; red—final field. The lava flows were sketched on the basis of ALI, Landsat and Sentinel imagery (USGS and ESA)

At Fimmvörðuháls, only stationary observations were made. Traverses were not possible due to the small plume and the location of the eruption. Traverse measurements were made for both Eyjafjallajökull summit and Holuhraun eruptions. A single stationary instrument was used at two locations over the four-day period at the Fimmvörðuháls fissure (Fig. 2a). A range of elevations over the vent were measured to determine changes in plume composition.

On 24 April 2010, spectra of the summit eruption plume were also collected from a fixed position, 12 km southeast of Eyjafjallajökull between 14:55 and 15:47 UT. The instrument box was mounted on a tripod and angled towards the plume NE of our position at ~ 25° from vertical. We estimate that the plume in the spectrometer’s field of view was 20 km downwind from the summit. Spectra were collected every 15 s, based on an optimal exposure time of 115 ms and 130 spectral averages for an improved signal to noise ratio.

At Holuhraun, stationary UV spectra were collected on 20 September 2014, again from a fixed position with fixed field of view between 10:00 and 16:00. This was a particularly clear day at the eruption site, and a map of the locations of the measurements is shown in Fig. 2b.

Traverse measurements were made at Eyjafjallajökull as follows: the instrument box was mounted on a vehicle, so the telescope viewed the zenith sky, and driven underneath the plume at a constant speed of ~ 70–80 km h^−1^. Before each traverse, the exposure time and the number of co-added spectra were selected according to the light conditions, so that a spectrum would be recorded every 3–4 s (corresponding to ~ 70 m distance along the road). Dark current, electronic offset and background (clear sky) spectra were collected at the beginning and end of each traverse, and all spectra collected during the traverses were time- and position-stamped using a USB GPS receiver.

On 23 April, five consecutive traverses (TR1 to TR5) were performed 45–70 km north of the summit crater between 10.41 and 14:54 UT. For these acquisitions, the optimal exposure time was 120 to 130 ms with 25 to 24 co-adds. An average of 790 ‘plume’ measurements were collected for each traverse. On 24 April two traverses (TR6 and TR7) were performed 10–30 km southeast of the crater between 09:55 and 11:03 UT. An exposure time of 140 ms was used with 25 co-adds, and ~ 470 plume data were recorded per traverse. Wind direction (and/or plume height) was highly variable during this period and there appeared to be a second branch of the plume travelling ENE, which we could not access due to icy roads. Between 17:46 and 18:58 UT, the plume direction shifted and we were able to perform a further three traverses (TR8 to TR10) 10–35 km west of the crater. For these traverses exposure times of 160 to 200 ms were optimal, with 22 to 18 co-adds. An average of 350 plume measurements were recorded per traverse. A map of the traverses is included as Figure A5.

At Holuhraun, traverses were performed using the methodology outlined in Pfeffer et al. (2018), where traverse data are presented. The methods used the same scripts as detailed above, with exposure time and co-adds selected prior to the traverse according to the light conditions. Measurements were made on 3, 4, 18, 19, 20 and 21 September 2014, and 21 and 22 January 2015. Many of these data are reported elsewhere (Burton et al. 2015; Ilyinskaya et al. 2017; Pfeffer et al. 2018). Here, we show some previously unpublished data: BrO measurements from stationary DOAS on 20 September that allowed a range of plume ages to be measured (See Fig. 1b); glass data from samples collected along the flow on the same day; and FTIR gas data from 18–21 September, focussed particularly on lava flow outgassing.

### Gas retrievals from UV spectra

All data were analysed using DOASIS software (Kraus 2006), with JScript’s available at http://www.geog.cam.ac.uk/research/projects/doasretrieval/. Each recorded spectrum was first corrected for electronic offset and dark current, and then shifted to give the best coincidence with a high-resolution solar emission spectrum (Kurucz 1995), convolved with a 0.45 nm FWHM Gaussian slit-function. Column amounts of gases were retrieved from each spectrum following standard differential optical absorption spectroscopy procedures (Platt 1994; Platt and Stutz 2008). During all fits a small shift (± 0.1 nm) and squeeze (0.99–1.01) of the absorption cross sections were allowed to account for optical misalignments and shift in the spectral calibration of the spectrometer (Stutz and Platt 1996).

For SO_2_, the fitting window 314–330 nm (traverses), 314–326 nm (stationary), was selected to minimise the dilution effect (Kern et al. 2009; Mori et al. 2006) and obtain a near-random fit residual structure with minimal standard deviation. High-resolution laboratory SO_2_ [at 273 K:(Bogumil et al. 2003)] and O_3_ [at 246 K: (Voigt et al. 2001)] absorption cross sections were convolved with the instrument line shape (FWHM = 0.45 nm) for use in the fitting procedure. A background spectrum (to account for the solar Fraunhofer lines), and a Ring spectrum (calculated from a solar spectrum (Kurucz 1995) using WinDOAS software, to account for the effect of rotational Raman scattering in the atmosphere), were also included in the fit, and a third order polynomial was used to remove broadband structures and the effects of Rayleigh and Mie scattering. A GPS was used to obtain the path of the traverse, and this was then combined with the gas data using calcflux software to calculate the SO_2_ flux. The plume speed required in the calculation of the fluxes was derived from wind speed observations at plume altitude obtained from the Icelandic Meteorological Office station closest to the site.

To analyse BrO, spectra were co-added in groups of five (stationary) or ten (traverse at Eyjafjallajökull, on top of original co-adds) to improve the signal-to-noise ratio. Retrievals were then performed in the spectral range 332–357 nm, which includes five BrO absorption bands and has minimal interference from the SO_2_ absorption feature discussed above. Absorption cross sections for BrO [at 273 K:(Fleischmann et al. 2004)], SO_2_ [at 273 K: (Bogumil et al. 2003)], O_3_ [at 246 K:(Voigt et al. 2001)], NO_2_ [at 246 K:(Vandaele et al. 2002)] and O_4_ (Greenblatt et al. 1990) were included in the fit, along with a background spectrum, a Ring spectrum and a third order polynomial. As a validation of our retrievals we also fitted BrO in the spectral ranges 327–353 nm (containing five BrO absorption bands) and 323–357 nm (containing seven BrO absorption bands). Very similar results were obtained.

Methods used in the retrievals at Holuhraun differed slightly because very high levels of SO_2_ resulted in total absorption at shorter wavelengths (310–326 nm), and therefore we selected the window 360–390 nm instead following Bobrowski et al. (2010).

All the measurements reported in this paper took place on sunny days with minimal cloud cover.

### OP-FTIR measurements

FTIR measurements were made during the Holuhraun eruption as reported by Pfeffer et al. (2018), and analysed for SO_2_, HCl, HF, CO, CO_2_, H_2_O, and OCS. Here, we report additional measurements made over the lava field (excluding the main plume from the field of view), using the same methods as detailed in Pfeffer et al. (2018). The spectrometer was pointed at an overflow on the side of the spatter cone, using the lava as a source of infrared. This allowed us to measure the gas content over the lava field. Measurements of the main plume used the lava fountains as the source (Pfeffer et al. 2018). The data were analysed using the code of Burton et al. (2007). Measurements of H_2_O and CO_2_ were corrected to account for the atmospheric contents of these species.

### Glass geochemistry

Fimmvörðuhals lava was sampled on 7 April 2010 from a recently cooled flow to the west of the fissure, and a sample of molten lava was taken from the active flow front and quenched. Tephra was also sampled from around the eruption site (see supplement). Recently cooled lavas were sampled at Holuhraun on 20 September 2014, at locations shown in Fig. 2d.

Thin sections were analysed using a Cameca SX100 electron microprobe at the University of Cambridge for major elements, S, Cl and F. For volatile elements, a 10 µm, 20 nA beam was used at 15 keV (major elements at 6 nA in a separate condition). Counting times were 90 s for Cl, and 60 s for S and F. See online Appendix for full analytical conditions and standards.

### Visual and audio observations (Fimmvörðuháls)

A Sony High Definition video camera (frame rate 25 fps) was used to film several episodes of fire fountain activity in the evolving fissure system at Fimmvörðuháls. This video was analysed qualitatively using Eagle Eye software, and semi-quantitatively by spectral analysis of the audio track. The audio was contaminated with extraneous noise from tourist helicopters and vehicles, and signal processing to remove the noise met with limited success because (i) the high sampling frequency (44100 Hz) was important for maintaining the shape of the signal, so downsampling was not an option and datasets were therefore very large; (ii) both high-pass and low-pass filters ran into similar issues and also failed to eliminate the noise. However, the explosions related to bubble bursts had sufficiently distinctive signals to allow their precise identification. Thus, it was possible to identify changes in the periodicity of audio data to identify explosions over the course of the campaign, and to compare them with periodicities evident in the gas data.

### Analysis of periodicities

Time series data were imported into MATLAB, and the wavelet toolbox was used to construct scalograms. Wavelet analysis allows the use of a moving window, and so is more useful for non-stationary timeseries than Fourier analysis (Ilanko et al. 2015; Pering et al. 2019). We used wavelet transforms to visualise the wavelet power spectra and identify periodicities. To look for corresponding periodicities in different gas species, we used wavelet coherence analysis, applying the code of Grinsted et al. (2004). High coherence implies a strong correlation between the gas species.

### Analysis of plume chemistry

We simulate the BrO/SO_2_ ratio in the Eyjafjallajökull plume using the *PlumeChem* model (Roberts et al. 2009). This model simulates the reactive halogen chemistry in volcanic plumes based on gas-phase (including photolytic) and heterogeneous (gas-aqueous) chemical reactions as the plume disperses into and mixes with the background atmosphere. *PlumeChem* has previously been used to simulate BrO/SO_2_ ratios in plumes from Soufrière Hills, Etna and Villarrica (Roberts et al. 2009; 2018), associated ozone depletion in Mt Redoubt plume (Kelly et al. 2013) and to constrain NO_x_ emissions at Erebus (Boichu et al. 2011). A sensitivity study (Roberts et al. 2014) highlights key parameters that impact the downwind BrO/SO_2_ are: (i) proportions of SO_2_:Cl:Br in the plume, (ii) plume aerosol loading, (iii) plume-air mixing rates, and (iv) an emission source containing ‘high-temperature’ radicals. These are only partially constrained by available observations for the Icelandic eruptions. We use molar SO_2_:Cl:Br of 1:0.65:0.001 based on Allard et al. (2011), and an iterative process for estimating Br, an aerosol surface area of 10^–11^ µm^2^/molec SO_2_ following Roberts et al. (2018), and prescribe mixing of the plume with air using the field-observed wind speed, gas flux and width of the Eyjafjallajokull plume. As per previous studies (e.g. Bobrowski et al. 2007, see Roberts et al. (2019) for a discussion of limitations) the HSC model was used to estimate radicals (e.g. HO, Br, Cl) in the emission source.

Table 1 summarises the methods used in each location.

**Table 1:** Analyses conducted at the different eruptions

	Fimmvörðuháls	Eyjafjallajökull	Holuhraun
Stationary DOAS (plume)	X	X	x
DOAS traverses (plume + lava)		X	x
FTIR (lava)			x
Visual camera	X		
Thermal camera	X (see online appendix)		
Glass chemistry	X		x
Snow chemistry	X (see online appendix)		
Tephra particle size	X (see online appendix)		

## Results: Fimmvörðuháls

### Nature of the activity: visual observations

The Fimmvörðuháls episode was characterised by minor lava fountain activity. The confined eruption area allowed us to study the vent processes in detail (this was not possible at Holuhraun due to field conditions). We took thermal images of the lava flows, some of which are in online Appendix A1 and AV1 and show the morphology of the relatively small lava field for this eruption. We include particle size distributions for tephra collected at several locations around the volcano, along with snow chemistry in the online Appendix (A2). This suggests that the strongest fire fountain activity was largely confined to the immediate vicinity of the craters, with large clasts being found dominantly in the SW and close to the fissures. Snow chemistry also showed some evidence of deposition of volcanic volatiles with sulphur concentrations particularly high in the downwind sector.

**Fig. 3:**
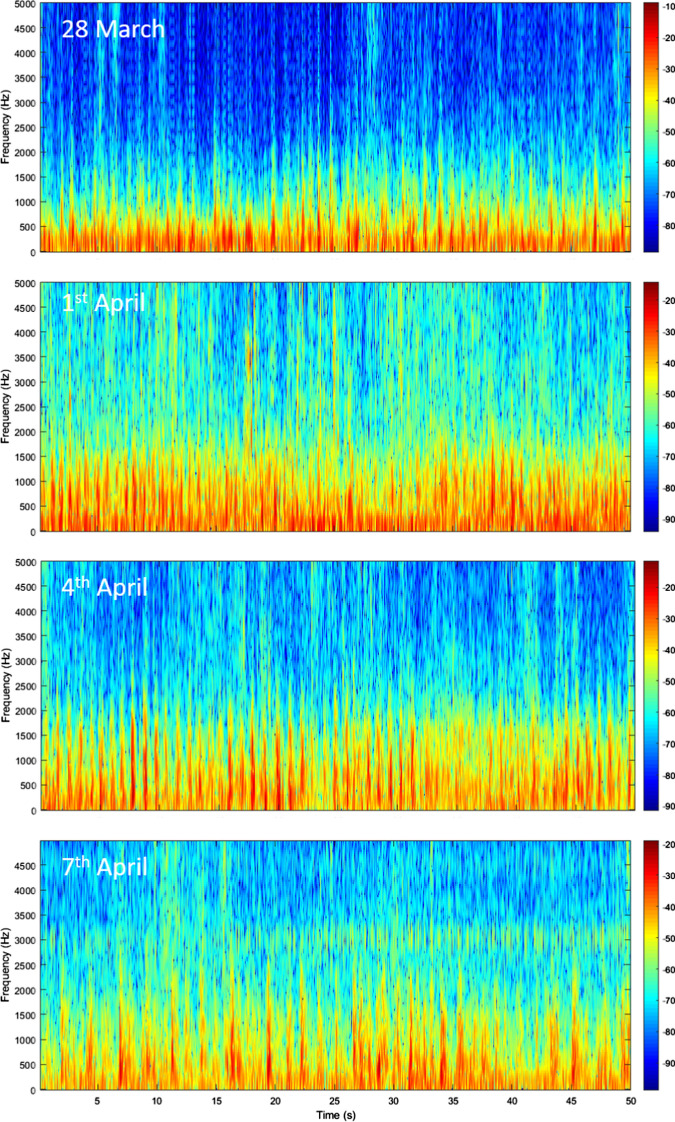
Spectrograms of acoustic signals from the most intense periods of fire fountain activity recorded at Fimmvörðuháls on each day (28 March, 1, 4 and 7 April). Colour bar shows power/frequency (dB/Hz)

Figure 3 compares 50 s intervals of processed audio recordings from each of the four days of observation. There were clear variations in the intensity of the fire fountain activity from day to day, with less frequent explosions occurring later in the eruption. Note that on 7 April, there was a lot of contamination of the signals from trucks and helicopters: this is partially visible at 3 kHz. Figure 3 demonstrates that activity peaked in the first few days of April, with strong and rapid bubble-burst activity from the fissure. By 7 April, the rate and intensity of the explosions were significantly reduced. However, there is clear periodicity in the explosions at some points in the data—for example, there was clear pulsatory activity at about 1 Hz on 4 April. Video footage suggests that the bubble-burst activity had become more distinct by this point because the fissure had fewer active vents and fewer bubble bursts were observed in each vent.

### Degassing processes and geochemistry

#### Results from stationary DOAS

**Fig. 4:**
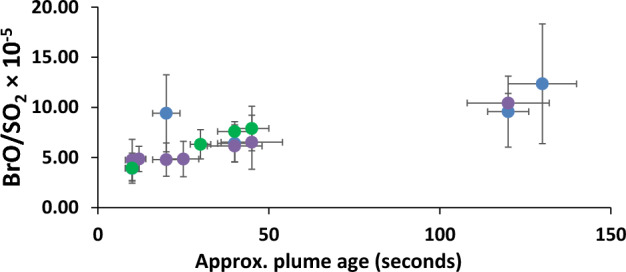
BrO/SO_2_ molar ratios in the young Fimmvörðuháls plume. Green: 4 April; dark blue: 7 April; purple: 1 April 2010. Average error on BrO (molec/cm^2^): 5 × 10^13^. Average error on SO_2_ (molec/cm^2^): 6 × 10.^17^

At Fimmvörðuháls, the very confined field conditions meant that traverses could not be made close to the fissures, and the very small and very young plume had to be measured in the near-field by rotating the spectrometer angle. This introduces some error on the column amounts due to differential scattering conditions, and the high winds on 28 March and 1 April. However, there was sufficient scope to identify an increase in BrO abundance as the plume moved away from the vent on three days (1, 4, 7 April) (Fig. 4). The data suggest that the BrO content of the plume generally increases with age, as more HBr is oxidised to BrO and due to interconversions of different reactive bromine species. The ratio measured in the more mature plume—1.01 × 10^−4^—is approaching that measured in the plume of the summit eruption (see below).

#### Periodicity

**Fig. 5:**
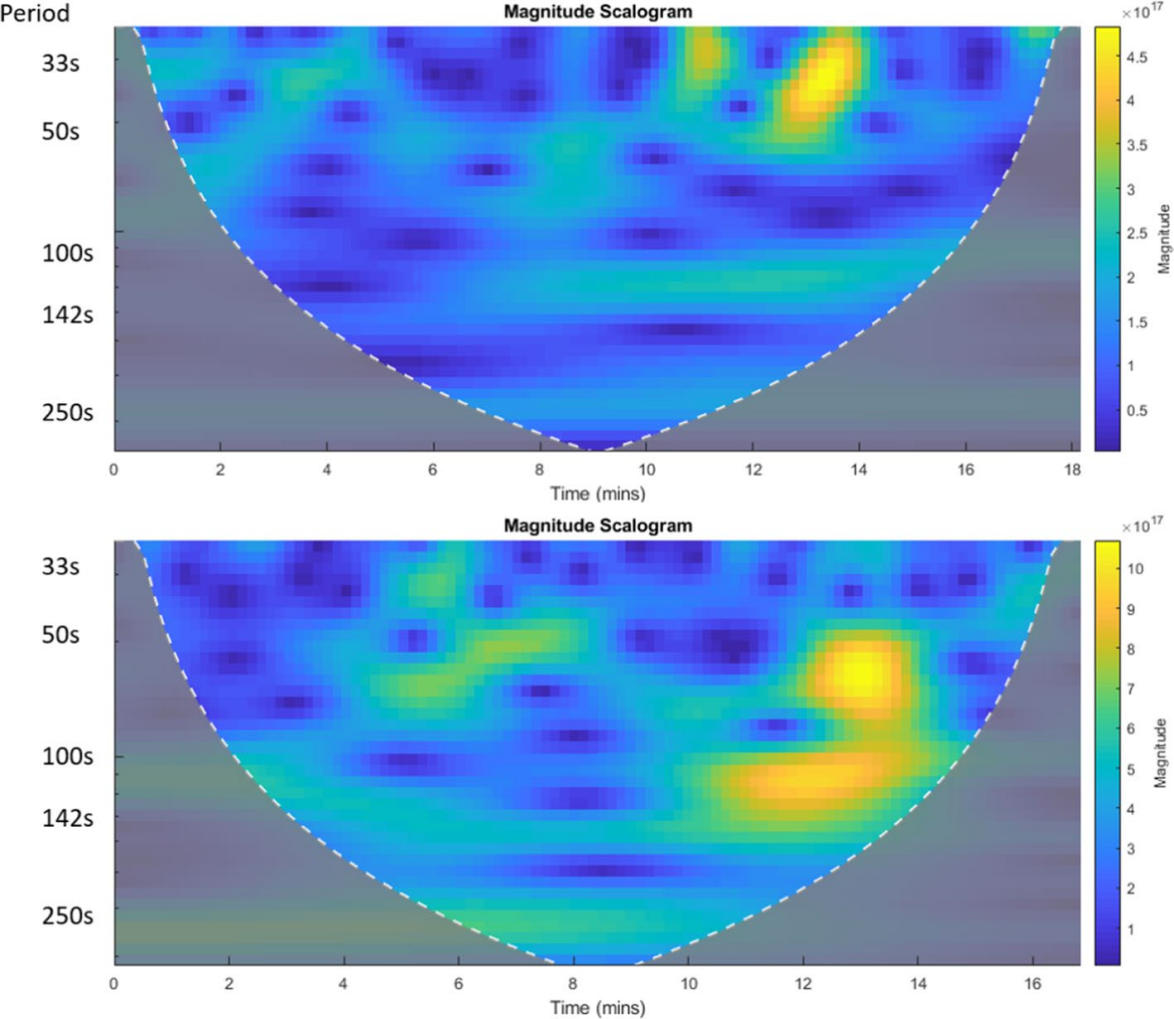
Two continuous wavelet transforms of DOAS SO_2_ column amounts at Fimmvörðuháls from 7 h April 2010 from 14:06 to 14:25 and 14:26 to 14:46, showing evidence of periodicity intermittently at 142 s (*Y*-axis shows the period) and at other periods to a lesser extent. Green to yellow areas show periods where the signal was periodic—some of this is intermittent and other parts are more persistent

Combining the SO_2_ column amount time series and acoustic data, we found qualitative relationships between the acoustic spectrograms and the (temporally displaced) DOAS SO_2_ data (Figure A3), and also some limited periodicity within the SO_2_ data itself. This is shown in Fig. 5 (at around 50 s and 140 s). The temporal resolution of the DOAS data does not allow identification of bubble bursts. The periodicity on 7 April, when the fire fountain activity was subdued, was the strongest in our data, perhaps suggesting that there was an intermittent supply of magma to the surface at this point in the eruption. In general, though, it is impossible to separate periodicity at source with periodicity due to plume transport processes (Woitischek et al. 2021).

#### Glass chemistry

We analysed glasses from the lava and tephra from this eruption. Lava samples were almost completely crystalline, with the exception of a quenched sample, which contained some glassy pools that allowed analysis of the glass composition. Representative tephra and lava glass compositions are provided in Table 2. Only sulphur was lower in the lava than the tephra, suggesting that it continued to degas in the lava flows. Cl and F were enriched in lava—though quite variable, with no discernible spatial pattern. Cl/F was relatively constant in the lava, with lower values than in the tephra due to higher F. Major element data are also significantly different between the tephra and the lava, which is expected because of extensive crystallisation in the lava flows (lower TiO_2_, FeOT, MgO, CaO and higher Na_2_O, K_2_O in the lava).

**Table 2:** Representative glass compositions (T = tephra; Q = quenched lava from flow front) at Fimmvörðuháls

	T1	T2	T3	T4	Q1	Q2	Q3	Q4
SiO_2_	48.97	47.82	47.70	47.27	54.02	53.71	54.38	54.48
TiO_2_	4.75	4.42	5.02	4.92	4.28	4.78	4.44	3.23
Al_2_O_3_	13.95	14.42	12.75	12.80	12.61	13.30	12.85	11.73
FeOT	13.54	12.69	14.80	14.21	10.14	11.66	9.92	10.25
MnO	0.32	0.18	0.29	0.26	0.26	0.25	0.37	0.20
MgO	4.38	4.59	4.90	4.95	2.85	2.27	2.87	5.47
CaO	9.72	10.59	10.22	10.12	5.65	4.54	5.58	8.44
Na_2_O	3.94	3.63	3.63	3.65	4.31	5.20	3.38	4.53
K_2_O	0.97	0.95	1.00	1.05	3.08	2.66	3.15	2.15
P_2_O_5_	0.58	0.61	0.67	0.63	1.77	1.49	1.74	1.26
S (ppm)	410	310	346	337	dl	dl	dl	dl
Cl (ppm)	896	548	621	563	886	820	1128	528
F (ppm)	964	1035	1492	870	3038	2656	3645	1630
S/Cl	0.46	0.56	0.56	0.60	0.02	0.00	0.03	0.12
S/F	0.43	0.30	0.23	0.39	0.01	0.00	0.01	0.04
Cl/F	0.93	0.53	0.42	0.65	0.29	0.31	0.31	0.32
Total	101.43	100.14	101.30	100.11	99.37	100.20	99.16	102.02

### Eyjafjallajökull summit eruption results

#### *SO*_*2*_* flux measurements*

A summary of SO_2_ flux estimates is given in Table 3. The average SO_2_ flux from five traverses measured on 23 April was 201 kg s^−1^ (equivalent to 17,400 t d-1). On 24 April, the average flux from 3 traverses was 118 kg s^−1^ (equivalent to 10,200 t d-1). We note that the variability in fluxes computed from individual traverses on both days can be primarily attributed to changing wind direction during traverse. On 23 April, the wind was changing from south easterly to southerly during the measurement period: we were travelling with the changing plume direction during SW-NE traverses (TR1, TR3, TR5) and against it during NE-SW traverses (TR2, TR4). On 24 April, the wind was changing from a northeasterly to an easterly direction during the measurement period: we were travelling with the changing plume direction for TR9, and against it for TR8 and TR10. The consistent pattern in the SO_2_ fluxes gives us confidence that the averages are meaningful, but the variability is not closely related to changing volcanic activity: the plume from the road was relatively old, changing direction and turbulent.

**Table 3:** SO_2_ emission rates calculated by ground-based UV spectroscopy on 23 and 24 April 2010 at Eyjafjallajökull summit eruption

Traverse number	Traverse start time (UT)	Traverse end time (UT)	Wind speed (m s^−1^)	Altitude (km)	SO_2_ flux (kg s^−1^)
23_TR1	10.51	11.37	5	3	219
23_TR2	11.44	12.14	5	3	147
23_TR3	12.25	13.15	5	3	214
23_TR4	13.24	13.58	5	3	172
23_TR5	14.13	14.54	5	3	252
24_TR6	10.02	10.31	–	–	–
24_TR7	10.36	11.02	–	–	–
24_TR8	17.47	18.05	5	2.5 to 3	114
24_TR9	18.08	18.35	5	2.5 to 3	132
24_TR10	18.38	18.56	5	2.5 to 3	108

#### BrO measurements

BrO was detected in traverse data (TR2 to TR10) performed on 23 and 24 April 2010, and in stationary data (S1) from 24 April 2010 (Table 4). We note that BrO was not detected in TR1 on 23 April since the spectral fits were of lower quality: the amplitude of the fit residuals from TR1 was significantly larger (1 × 10^−2^ molec/cm^2^) than from other traverse data (typically 4 × 10^−3^ molec/cm^−2^). Considering the maximum peak-to-peak amplitude of the BrO differential cross section, ~ 2 × 10^−17^ cm^2^ molecules^−1^, we would need at least 5 × 10^14^ molecules cm^−2^ of BrO in the measured column for it to be detected in spectra from TR1. The maximum column amount of BrO measured from other traverse and stationary datasets, however, was 4 × 10^14^ molecules cm^−2^, that could be detected given the lower amplitude of residual noise in the data (4 × 10^−3^).

**Table 4:** Summary of BrO/SO_2_ molar ratios from traverse (TR) and stationary (S) data from Eyjafjallajökull summit, shown in chronological order (n.d. = not detected). The plume age is based on a wind speed of 5 m s^−1^ for all data

Experiment number	Time (UT)	Max SO_2_ (molec cm^−2^)	Distance from summit (km)	Plume age (hours)	BrO/SO_2_
23_TR1	10.51–11.37	1.70 × 10^18^	45–55	2.5–3	BrO n.d
23_TR2	11.44–12.14	1.32 × 10^18^	45–55	2.5–3	0.9 × 10^–4^
23_TR3	12.25–13.14	2.17 × 10^18^	45–65	2.5–3.5	0.8 × 10^–4^
23_TR4	13.24–13.58	1.30 × 10^18^	45–65	2.5–3.5	0.8 × 10^–4^
23_TR5	14.13–14.54	1.65 × 10^18^	45–70	2.5–4	1.3 × 10^–4^
24_TR6	09.54–10.32	1.52 × 10^18^	10–30	0.5–1.5	2.4 × 10^–4^
24_TR7	10.33–11.03	1.25 × 10^18^	10–30	0.5–1.5	1.9 × 10^–4^
24_S1	14.55–15.47	8.12 × 10^17^	20	~ 1	1.5 × 10^–4^
24_TR8	17.46–18.05	2.66 × 10^18^	10–25	0.5–1.5	1.6 × 10^–4^
24_TR9	18.08–18.36	1.91 × 10^18^	10–35	0.5–1.5	1.7 × 10^–4^
24_TR10	18.37–18.58	2.06 × 10^18^	15–35	1–2	1.7 × 10^–4^

**Fig. 6:**
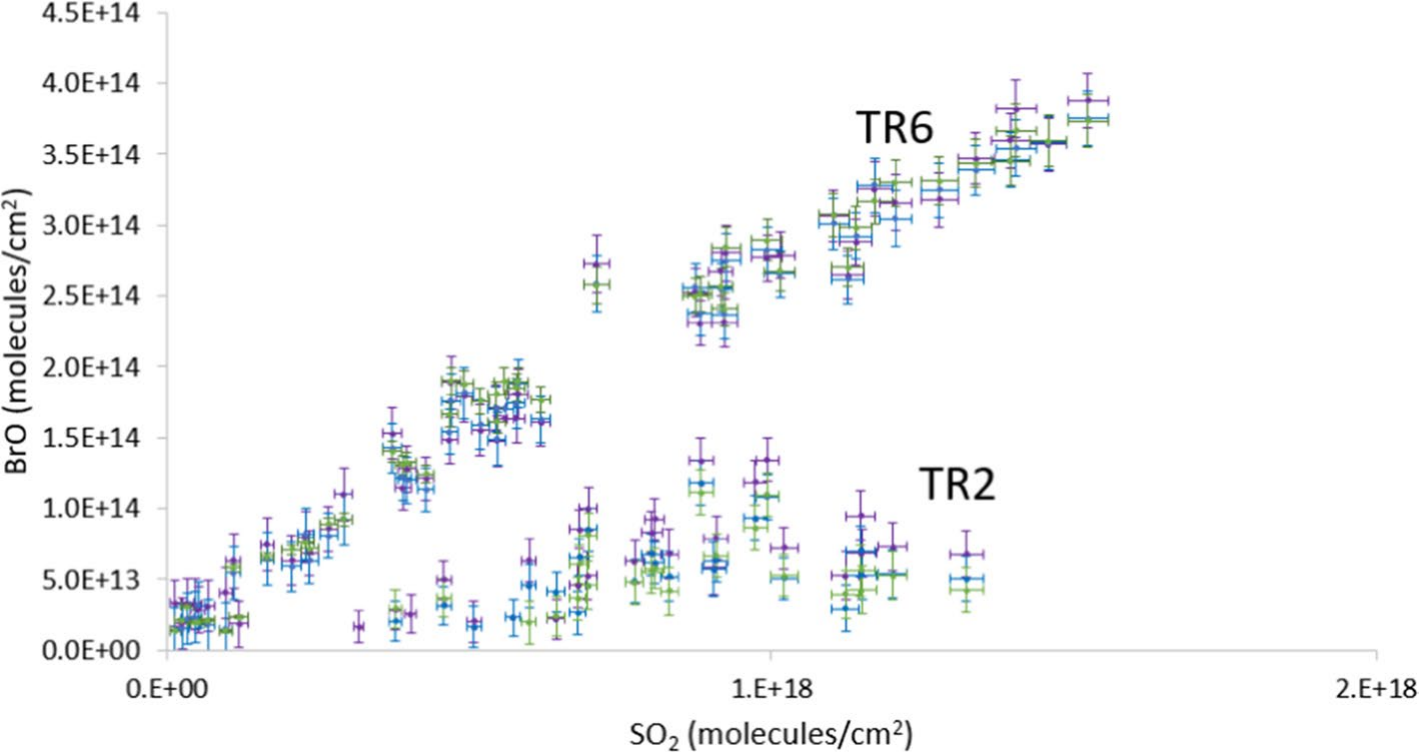
Column amounts of BrO vs. SO_2_ measured during TR2 on 23 April 2010 at Eyjafjallajökull summit (2.5–3 h plume age), average BrO/SO_2_ = 7.3 × 10^−5^; and TR6 on 24 April 2010 (0.5–1.5 h plume age), average BrO/SO_2_ = 3.6 × 10^−4^. Results from three BrO spectral fitting windows are shown: the 332–357 nm window used for final results in purple, 327–353 nm in blue and 323–357 nm in green

Example plots of BrO vs. SO_2_ for TR2 (45–55 km downwind) and TR6 (10–30 km downwind) are shown in Fig. 6. A summary of BrO/SO_2_ ratios from all traverses is shown in Table 4. The average BrO/SO_2_ ratio from four traverses (TR2–5) performed between 45 and 70 km downwind of the summit on 23 April is 1.0 × 10^−4^. The average from measurements (TR6–10) made between 10 and 35 km downwind on 24 April is 2.0 × 10^−4^.

#### Results from Holuhraun

At Holuhraun, the primary techniques used were stationary and mobile DOAS and OP-FTIR. Glass and melt inclusion chemistry were also measured for selected samples. The mobile DOAS and main vent FTIR data are published elsewhere (Pfeffer et al. 2018), but will be discussed in the context of this paper here, particularly focussing on the outgassing of lava flows and the halogen content. The stationary DOAS involved measurement of BrO/SO_2_ ratios. Video of fire fountain activity was also taken and analysed, but it was not possible to extract records of bubble bursts from the video. This was due to a distinct difference between the type of activity here and that at Fimmvörðuháls. At Holuhraun, the activity was characterised by churning of a lava lake and was considerably more chaotic than the gas pistoning activity that was observed at Fimmvörðuháls. This is consistent with the much higher eruption rate at Holuhraun (Pedersen et al. 2017).

#### Halogen emissions

**Fig. 7:**
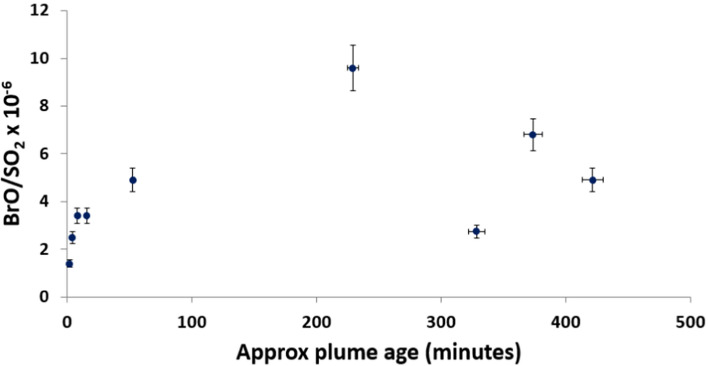
Shows the evolution of BrO in the plume along the lava flow at Holuhraun on 20 September 2014. The size of the eruption and the field conditions on 20 September allowed this detailed analysis—it was possible to drive along the flow for ~ 20 km and take stationary measurements at a range of plume ages (plume age was estimated from video footage with an error of about 15 min)

The BrO/SO_2_ ratio at Holuhraun (4.4 × 10^−6^) was considerably lower than that at Fimmvörðuháls—entirely because the sulphur content was much higher. The column amounts of BrO were 1.5 to 7.0 × 10^14^ molecules/cm^2^, comparable to those at Fimmvörðuháls. Since the eruption site at Holuhraun was more accessible and the plume much more extensive, it was also relatively straightforward to make measurements of plume of different ages. Figure 7 shows the evolution of BrO/SO_2_ against approximate plume age (estimated from video footage).

**Fig. 8:**
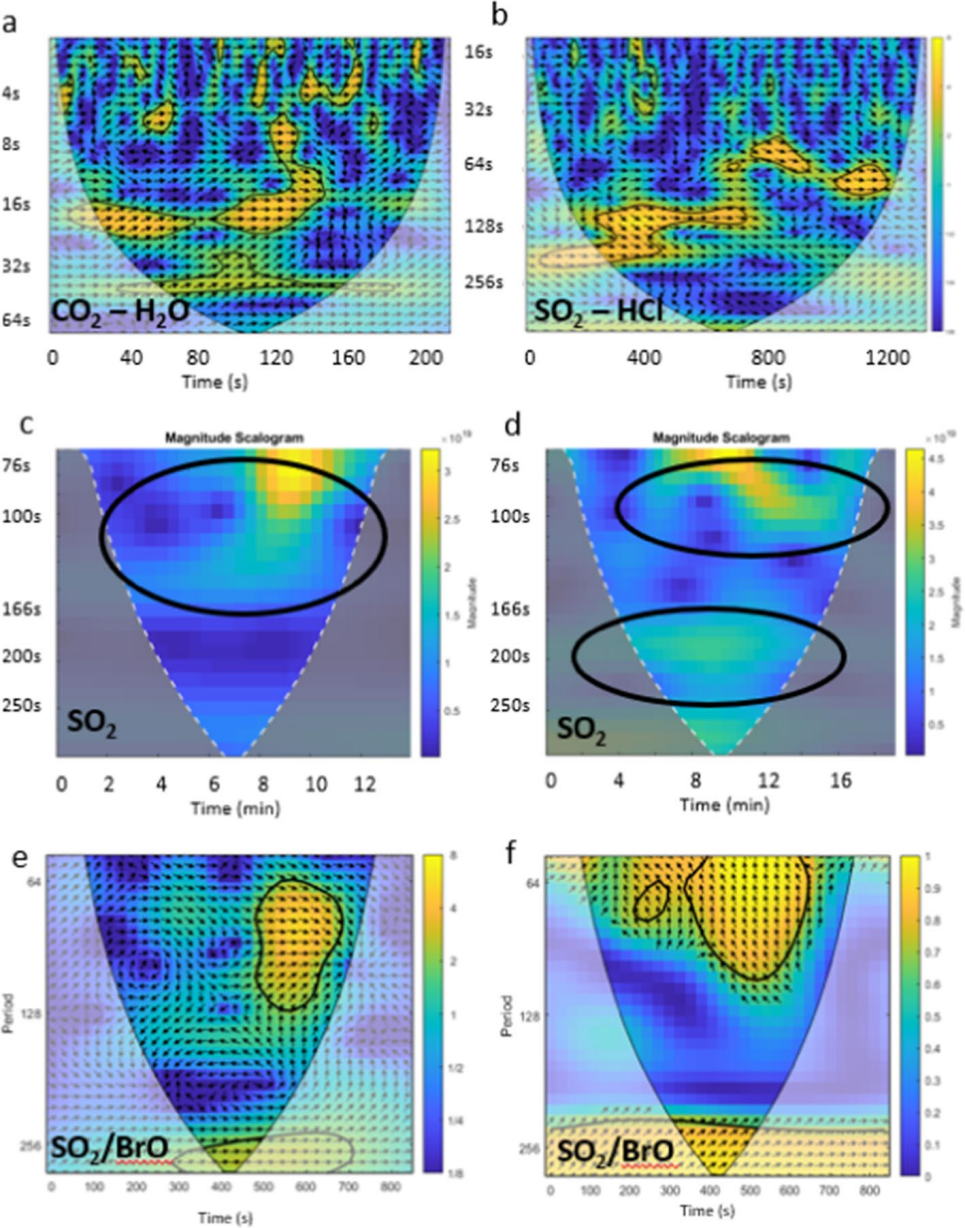
Wavelet scalograms for FTIR and DOAS column amounts from Holuhraun, with period on the y-axis in all plots. **a** and **b** Show wavelet coherence plots (showing periodicities common between the datasets; Grinsted et al. 2004) of FTIR measurements at Holuhraun on 19 September, showing several intermittent areas of coherence between the gases. **c** and **d** Show wavelet scalograms of DOAS SO_2_ data from Holuhraun close to the vent on 20 September, showing some short-lived periodicity and weaker periodicity at higher periods. **e** and **f** Show wavelet coherence plots between DOAS SO_2_ and BrO on 20 September (Grinsted et al. 2004) with arrows showing the relative phase relationship between the two gases—arrows pointing right are in phase, left anti-phase, down SO_2_ leads BrO by 90° and up BrO leads SO_2_ by 90°. The black circles highlight the region that corresponds across the datasets. The colour bar in coherence plots shows the correlation strength

**Fig. 9:**
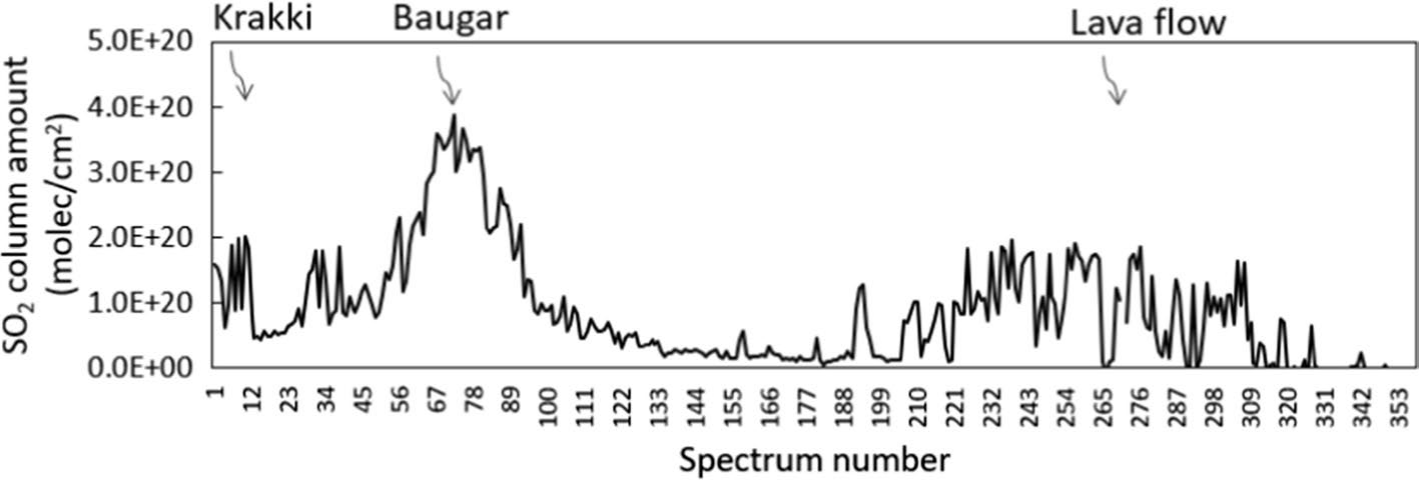
Traverse along the Holuhraun fissure, showing the outgassing from the lava field. Krakki is a small vent to the south of the fissure. Baugar was the main vent. Spectrum number denotes time (one spectrum per 5 s)

We used wavelets to examine periodicity in the time series of SO_2_ and BrO column amounts (those collected from stationary positions close to source) and also in the FTIR data timeseries of column amounts reported by Pfeffer et al. (2018) from the main vent (Fig. 9 a, b). The DOAS results are shown in Fig. 8 c–f. The DOAS data suggest a weak periodicity in the gas emission at around 111–143 s, present in both sets of data, and intermittent, stronger periods at around 77–91 s. Other intermittent periodicities are also apparent in the data.

#### Lava field degassing

During the field campaign, several traverses were made along the lava flow, with the UV spectrometer’s telescope horizontal to capture, semiquantitatively, the plume coming from the lava field (only semi-quantitative due to the high error from the telescope angle). An example is shown in Fig. 9. The measurements clearly show that there was substantial degassing of SO_2_ from the lava field—in some traverses, this was up to 20% of the total emission—more typically, it was around 5%. These measurements were taken when the lava field was highly active in September 2014; there was a fast-moving stream of lava down the centre of the field, with lobes to both sides (Fig. 2d).

FTIR spectra were also collected with the telescope trained on a hot part of the scoria cone (constantly over-spilling) and hence with an atmospheric path above the lava flow field. Figure A4 shows plots of the column amounts of SO_2_, HCl and HF. A notable difference between the main vent record and the spatter cone data is that the lava field gas was richer in SO_2_ relative to CO_2_ and H_2_O (Table 5). In addition, HCl over the lava field was much higher than in the main plume (assumed to dominate the main vent measurements), and the S/Cl ratio was slightly lower.

**Table 5:** Comparison of gas ratios (with standard deviation) between the main vent and the lava flow at Holuhraun. The standard deviation is high due to gusting winds

	Main vent (*n* = 1118)	Lava field (*n* = 502)
SO_2_/HCl	70 (30)	46 (40)
HCl/HF	2.1 (1.6)	6.5 (2.6)
CO_2_/SO_2_	2.9 (1.5)	1.3 (0.8)
SO_2_/HF	140 (99)	231 (57)
H_2_O/SO_2_	21 (12)	12 (6.6)
S/Cl	36 (15)	23 (20)
Cl/F	2.2 (1.7)	6.6 (2.6)

#### Comparisons with petrological data

**Fig. 10:**
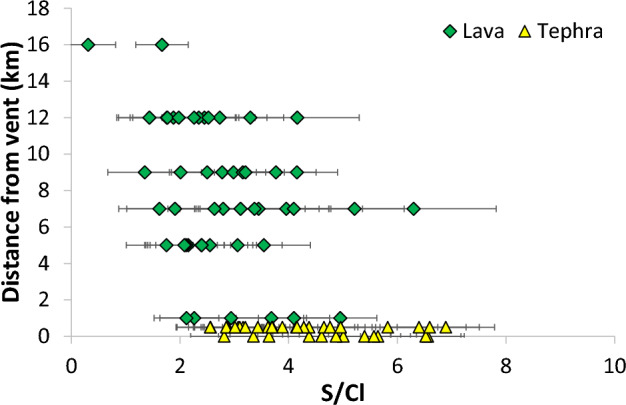
Glass S/Cl ratios in Holuhraun lava sampled sequentially along the flow, compared to tephra samples. Lava samples are green diamonds and tephra are yellow triangles

For Holuhraun, we used new glass data (see supplement) to look for variations in the S/Cl ratio in the lava flow field. Figure 10 shows the variation in S/Cl along the lava flow field in samples collected in September 2014. It shows that while the tephra show a wide range of S/Cl ratios (consistent with the data from other authors; Bali et al. 2018; Halldórsson et al. 2018), the lava samples consistently showed lower S/Cl ratios, and those furthest from the vent, the lowest ratios of all—though these were highly crystalline and hard to measure. The difference between the lava and the tephra is statistically significant (*t* = 5.88, *p* < 0.001). This might suggest that there was some Cl degassing from the lava flows, consistent with the gas measurements, but that the gas phase is still dominated by SO_2_ (see Fig. 9 and Table 5). However, the variation is only just outside of the error on the measurements. Petrolog modelling (see supplement) shows that Fe–Ti oxides stabilised in the flows, consistent with petrography (e.g. Figure A6) (Danyushevsky and Plechov 2011), and we note that the tephra were very glassy with few crystals, mainly of plagioclase—much crystallisation took place in the flows. Both Cl and particularly F content is considerably higher in lava samples than in tephras.

## Discussion

### Vent processes in the fissure eruptions

Fire fountain dynamics differed visually between the Fimmvörðuháls and Holuhraun eruptions. At Fimmvörðuháls, the proximity of the vents to the observation sites allowed the recording of audio-visual records that could not be obtained at Holuhraun due to the size of the lava field. We examined acoustic signals alongside our gas data for Fimmvörðuháls, and identified some evidence of semi-periodic activity, which we attribute to bubble bursting in the case of acoustic data. Periodicities in the gas column amounts are harder to explain on the basis of the period alone (periods are around 100–140 s), but are found in both FTIR and DOAS data. Throughout the sound files, there were periods of increased activity and other periods of diminished activity, some of which do correlate (coarsely) with the gas periods (see supplementary data), and this might suggest that there is a periodicity in gas supply and foam breakdown into slugs. However, the intermittent periodicities in the gas may also represent processes in the volcanic plume, such as interaction with ambient air and wind patterns or turbulence (Woitischek et al. 2021).

The basalts that were feeding the eruptions at Eyjafjallajökull and Holuhraun exhibit key differences that are well documented both in composition and in eruptive volume (Bali et al. 2018; Hartley et al. 2018; Moune et al. 2012; Sigmarsson et al. 2011). Our multidisciplinary datasets reinforce previous arguments that the magma feeding the Bárðarbunga eruption at Holuhraun was from a deeper source and richer in sulphur relative to halogens compared to that feeding Eyjafjallajökull and erupted at Fimmvörðuháls.

There are substantial differences between the two fissure eruptions in terms of degassing, too. The most obvious is magnitude: the Holuhraun eruption was substantially more voluminous, and produced much more sulphur dioxide (Bali et al. 2018; Halldórsson et al. 2018; Pfeffer et al. 2018; Schmidt et al. 2015). Likely as a result of the higher sulphur content, the BrO/SO_2_ ratio was much lower. BrO column amounts for similar plume ages were higher than those at Fimmvörðuháls, but by very little, despite the much higher emission rate. At Fimmvörðuháls, BrO was surprisingly high for a small volume eruption, however—particularly compared with Holuhraun (which involved larger magma volumes). This is consistent with halogen emissions recorded by Allard et al. (2011) in the plume for the summit eruption, which showed relatively high HCl emissions (0.13 mol%) in the plume for the summit eruption. The relatively long residence time of the magma (Moune et al. 2012; Pankhurst et al. 2018) with opportunity to evolve led to halogen-rich, sulphur-poor emissions from Fimmvörðuháls.

Experimental studies on both chlorine and bromine partitioning have demonstrated that the behaviour of the halogens under magma chamber to vent to flow conditions is highly complex, and depends on magma and co-existing fluid phase compositions, temperature and redox conditions (Aiuppa et al. 2009; Alletti et al. 2009; Cadoux et al. 2018; Webster et al. 2009). However, Cadoux et al. (2018) produce a preliminary model for S–Cl–Br degassing in a basaltic system. Their model suggests that such systems should move from an initially S-rich melt and gas towards a relatively Cl–Br rich melt and gas, as is also suggested by our data, particularly from Fimmvörðuháls. Furthermore, recent studies suggest that Cl solubility in the melt is strongly dependent on temperature, with decreasing temperature in mafic systems leading to decreasing Cl solubility (Cassidy et al. 2022; Thomas and Wood 2021), consistent with some HCl degassing from the flows. Cassidy et al (2022) further note that the partitioning of halogen species is strongly compositionally dependent, with their experimental measurements of *D*_Br_^f/m^ ranging from 7.1 + / − 6.4 in basaltic andesite to 31.3 + / − 20.9 in dacite. They also note that Br is more sensitive to temperature than Cl in these more silicic systems. The complexity of untangling the intrinsic variables that control partitioning, coupled with the lack of capability to measure HBr directly, makes it challenging to infer any quantitative estimate for initial magma Br contents from our data.

We note that flux measurements are very strongly dependent on wind speed, and in our case may be considerably higher than we report because of fluctuations in the wind speed and the high sensitivity of the flux calculation to small changes in wind speed—some of the flux measurements for individual traverses were in excess of 100,000 t/d SO_2_. There is considerable temporal variation throughout the day in SO_2_ emission in general and care should be taken in interpreting daily values, particularly as the time resolution of spectra and of the modelled wind speed is different between studies. We add some new traverse data from Eyjafjallajökull, which suggest SO_2_ fluxes of up to 21,800 tonnes per day, which is much lower than those from Holuhraun.

### Lava field degassing at Holuhraun and the Moðuharðindin

At Holuhraun, there was significant degassing of the lava field—particularly of SO_2_, which was detectable in traverses. Holuhraun has widely been regarded as a small-scale analogue for the Laki eruption in 1783-4 (Schmidt et al. 2015), and the presence of considerable gas at a lower level in the troposphere is consistent with reports from Laki of the “mist hardships”—a low-level volcanic cloud (Simmons et al. 2017). During the Holuhraun eruption, the civil defence maintained an exclusion zone as a result of the gas hazard in the proximal area, and the DOAS data back up the need for such a zone: while there were significant variations in the SO_2_ emissions from the eruption, anyone in close proximity to the vents or active flows could have been exposed to SO_2_ concentrations close to the WHO recommended limits (40 μg/m^3^).

Our FTIR data for Holuhraun, collected using the side of the spatter cone as an IR source (where there was constant lava input), suggest that the plume over the lava field was richer in HCl relative to SO_2_ compared with the plume from the main vent. The glass data show higher S/Cl in the tephra than the lava, suggesting that S degassing was still dominating in the flows and that the difference in the main vent gas chemistry is likely due to fluxing of SO_2_ from depth (where degassing of HCl is suppressed). We note, though, that we have not measured H_2_S emissions and the role of sulphides in this magma is still unclear (Sigmarsson et al. 2020; Gauthier et al. 2016). Higher HCl degassing at the surface would be consistent with other volcanic systems, and it could potentially be driven by crystallisation increasing the Cl concentration in the melt (Beermann et al. 2015; Edmonds et al. 2001; Humphreys et al. 2009; Unni and Schilling 1978) as well as by the effect of intrinsic variables on the partitioning of Cl between fluid and melt as discussed above.

In contrast, SO_2_/HF was very high over the lava field, suggesting that HF does not behave in a similar fashion to HCl under the conditions at Holuhraun and was not significantly degassing in the flows syn-eruption—this is consistent with other measurements made of gas around the eruption site that likely reflect a combination of the main plume and the lava field emissions (Gíslason et al. 2015; Ilyinskaya et al. 2017; Stefánsson et al. 2017), and with the HCl/HF ratios. It is also consistent with recent work on F partitioning (Cassidy et al. 2022). These results are in contrast to the filter pack measurements report in Sigmarsson et al.(2020) who found that SO_2_/HF was much lower (two orders of magnitude) in the residual gas from the lava field (after the eruption finished) than during the eruption. While the measurements have high errors, this might suggest that HF emission increased under post-eruptive conditions, with lower temperatures and when the melt would have been higher in F due to crystallisation. Indeed, the tephra samples had F contents largely below the detection limit (supplementary data), while lavas were higher in F. Alternatively, the melt may have been almost completely degassed in SO_2_ at the time of the post-eruptive measurements in Sigmarsson et al (2020), as they suggest.

Lava field degassing is a significant finding for risk assessment in Iceland, because the gases that are emitted from lava flows can present a near-field hazard. It also tallies with the findings of Thordarson and Self (2003) that there was likely a localised haze that resulted from near-field degassing during the Laki eruptions. It was likely highly variable during the course of the Holuhraun eruption, since the areal extent of the lava surface was also highly variable. Our data suggest that SO_2_ and HCl, with relatively less HF, were emitted from the Holuhraun flows syn-eruption, and that hazards from halogen gases are greater in the near field during the eruption (though slightly below hazardous levels as defined by the WHO). While halogens such as chlorine and fluorine appear to be even more enriched post-eruption relative to sulphur (Sigmarsson et al. 2020), the actual absolute gas emission may be significantly less.

### Halogen differences between volcanic systems

Both the volcanoes studied in this paper produced measurable BrO, though the Holuhraun eruption column amounts were typically lower than those at Eyjafjallajökull (both phases of the eruption, the summit data comparing well with the satellite measurements by Heue et al. 2011; Hormann et al. 2013). This is consistent with halogen emissions reported elsewhere for these eruptions (Allard et al. 2011; Pfeffer et al. 2018). However, heterogeneity in Cl isotopes and in F across Iceland suggests that there may be more complex origins for these variations in Iceland (Halldórsson et al. 2016; Rowe and Schilling 1979; Unni and Schilling 1978).

In both cases (Eyjafjallajökull and Holuhraun), the gas ratios detected at the surface during the eruption are products primarily of shallow processes rather than mantle source conditions. Thus, the emissions of the eruptions of Eyjafjallajökull were likely enriched in halogens relative to SO_2_ due to depletion of SO_2_, H_2_O and CO_2_ during shallow degassing while some of the magma was resident in sills; whereas, at Holuhraun it is primarily CO_2_ that was shallowly degassed while the magma travelled along the dyke towards the eruption site and SO_2_ and H_2_O degassed relatively little prior to arrival at the surface (Gíslason et al. 2015; Pfeffer et al. 2018); Bali et al (2018) estimate SO_2_ degassing commenced at around 3.7 km deep. The lava at Holuhraun has been shown petrologically to be derived from a well-mixed reservoir (Halldórsson et al. 2018), but these studies show that this is likely deeper in the crust, where halogen exsolution is inhibited—and sulphur saturation occurred late (Bali et al. 2018; Halldórsson et al. 2018). These studies also suggest that the higher sulphur contents of the Holuhraun lava are also consistent with the source being further from the Iceland mantle plume and more representative of MORB, with higher melt fraction (Bali et al. 2018; Burton et al. 2015; Halldórsson et al. 2018; Hartley et al. 2018; Moune et al. 2012).

Halogen behaviour between tephra and lava at Fimmvörðuháls were different to Holuhraun. The lava did not contain enough sulphur to calculate ratios reliably, but the Cl/F ratio in Fimmvörðuháls tephra (0.65) was much higher than in the quenched lava sample (0.31). At Holuhraun, F in the tephra samples was frequently below the detection limit, but where it was not, tephra and lava exhibited similar Cl/F ratios (0.26 for tephra and 0.23 for lava)—with lava containing appreciably higher absolute values for both Cl and F. This suggests that HCl in particular was able to degas rather more efficiently at Fimmvörðuháls than HF. At Holuhraun, there was some HCl degassing in the flows and HF degassing was relatively suppressed, as noted above.

Thus, the petrological literature suggests that the higher sulphur content at Holuhraun relates to deep processes (Bali et al. 2018; Hartley et al. 2018), and the gas data here and in the literature suggest that the halogen variations between volcanic systems most likely relate to different shallow degassing processes and their relationship to halogen partitioning between fluid and melt, as suggested by existing degassing models (Cadoux et al. 2018) and discussed above. However, we cannot address differences in halogens at source in this work and note that there is evidence of heterogeneity in Cl across Iceland (Halldórsson et al. 2016; see also Bali et al. 2018).

### Atmospheric processes: bromine chemistry

**Fig. 11:**
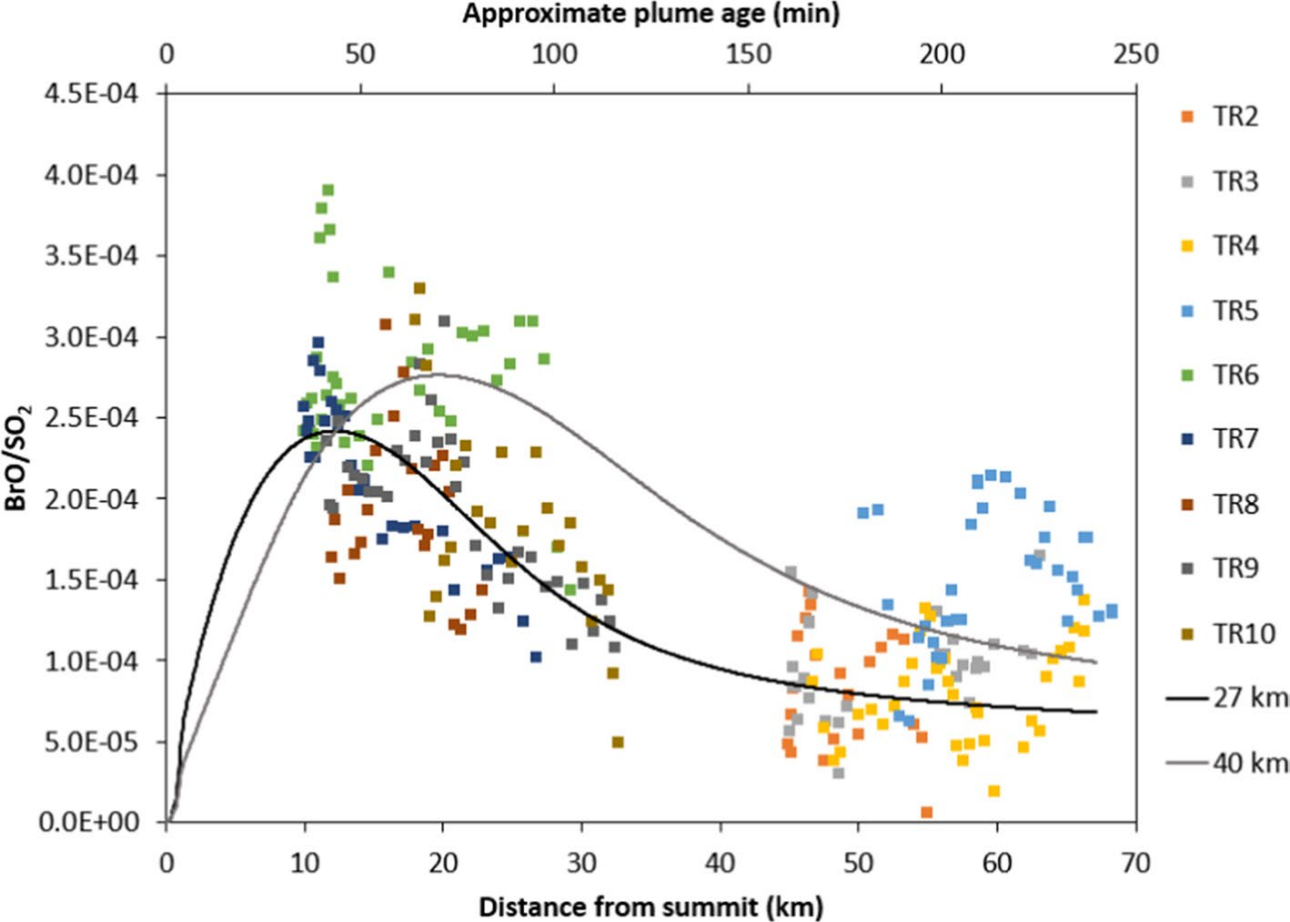
BrO/SO_2_ vs. distance from summit for the summit eruption of Eyjafjallajökull. Only points where the column amount of SO_2_ exceeds 5 × 10^17^ molecules cm^−2^ have been included so as to avoid large fluctuations in ratios caused by low column amounts of gas (and plume edge effects on the chemistry). The black and grey solid lines show model simulations for HBr/SO_2_ emissions of 4 × 10^–4^ for a plume that is 27 km wide at 20 km downwind and one that is 40 km wide at 20 km downwind, respectively

There is in all our datasets a distinct peak in the BrO/SO_2_ ratio in the early plume. This then dissipates slowly at a rate that depends largely on plume size at both the summit eruption of Eyjafjallajokull and at Holuhraun (the plume at Fimmvörðuháls was only measured very close to the vent and shows only the bromine explosion close to the vent). The BrO/SO_2_ ratios evolve in the plume in a consistent way for all the Eyjafjallajökull traverses, reflecting a consistent plume chemical evolution. Simulations using the PlumeChem model broadly reproduce the observed BrO/SO_2_ trend (Fig. 11), and provide further insight to the underlying chemistry.

**Fig. 12:**
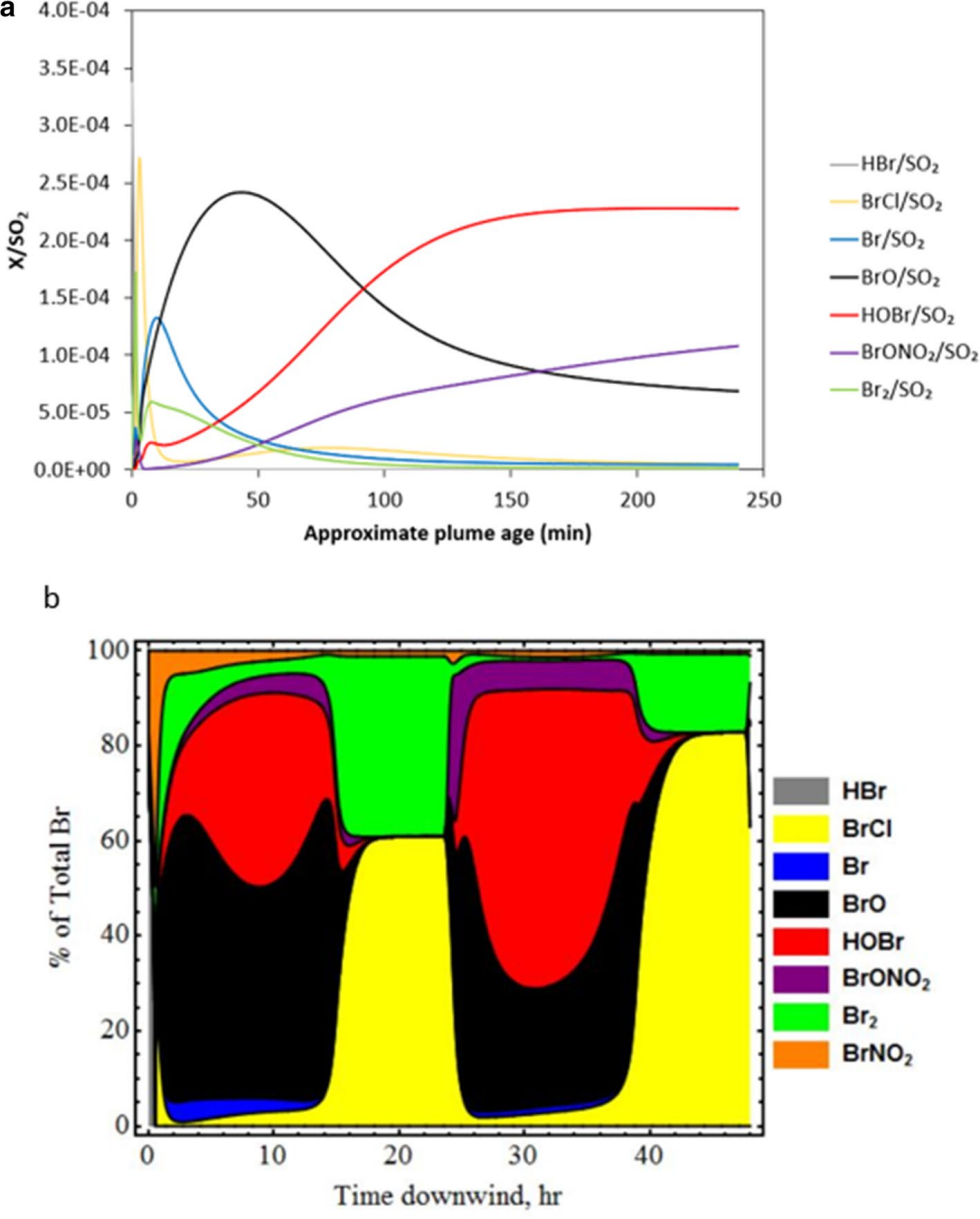
Speciation of reactive bromine in the Eyjafjallajökull summit plume vs. distance downwind from the summit **a** in the short term and **b** over 48 h. The simulation (**a**) is based on a HBr:SO_2_ of 4 × 10^−4^ for a plume that is 27 km wide at 20 km downwind (i.e. the black solid line in Fig. 12). The simulation (**b**) illustrates the halogen speciation for a plume dispersion over greater time- and regional scales. Gases HBr, BrO, Br, BrONO_2_, BrCl, Br_2_ and HOBr are shown as a ratio to SO_2_, which represents a plume tracer

BrO is formed by the “bromine explosion” in volcanic plumes (by which BrO is created from a multiphase and photolytic chemical reaction cycle, as described in the introduction), but the decline in BrO/SO_2_ further downwind does not reflect a ‘switching off’ of this mechanism. Rather, the model simulations suggest the observed decrease in BrO/SO_2_ (Fig. 11) is due to interconversion of reactive halogen species in the plume. Figure 12 shows the concentrations of BrO, HOBr, Br, BrCl, BrONO_2_, Br_2_ and HBr relative to plume tracer SO_2_, over a 4 h (70 km) simulation. The decline in BrO/SO_2_ beyond 10 km downwind is accompanied by a rise in HOBr/SO_2_ and BrONO_2_/SO_2_. This change in plume composition is due to entrainment of background air containing HO_2_ and NO_2_, promoting formation of HOBr and BrONO_2_. At the same time, the plume dispersion dilutes the absolute concentrations of aerosol and reactive bromine, slowing heterogeneous loss of HOBr and BrONO_2_. Therefore HOBr and BrONO_2_ become reservoirs for BrO in the downwind plume, as the plume chemistry evolves.

Extending *PlumeChem* simulations to 48 h shows a diurnal trend in the BrO/SO_2_ ratio (Fig. 12b). After sunset, BrCl and Br_2_ can build up in the plume due to their heterogeneous formation from HOBr and BrONO_2_ and the absence of photolysis. When sunlight returns, the “bromine explosion” can restart, if concentrations of critical plume components (e.g. reactive bromine, acid aerosol) are sufficiently high. This process is consistent with observations by Heue et al. (2010), who measured column amounts of SO_2_ on the order of 10^17^ molecules cm^−2^ and a BrO/SO_2_ ratio of ~ 1.3 × 10^–4^ just after 10:00 h UTC on 16 April 2010, in the plume aged 34–53 h downwind from Eyjafjallajökull. It is interesting to note the similarity between these values and our BrO/SO_2_ ratios in the plume < 70 km downwind. Whilst the ash-rich plume measured by Heue et al. (2011) (emitted from the volcano on 14 April 2010, at the start of the summit eruption) may not be directly comparable with our measurements made during an ash-poor phase of the eruption, the observation provides an illustration of the long effective lifetime of reactive bromine in volcanic plumes.

### Implications for monitoring of Icelandic fissure eruptions

The Icelandic Meteorological Office has an advanced volcano monitoring programme in Iceland, using high resolution spectrometers for scanning DOAS applications as part of the NOVAC network. Ultraviolet spectroscopy in Iceland is challenging between September and May because of the relative paucity of UV radiation in these months. Measurement of halogen species under low levels of UV is particularly challenging because of the low levels of the parent halogen species in Icelandic magmas, but there is clearly considerable variety in the content of halogens in Icelandic basalt and further work on this would be useful and is underway (Waters 2022). OP-FTIR combined with DOAS has the potential to improve the resolution of halogen measurements in Iceland. The models discussed above demonstrate that photolysis of Br_2_ is fairly efficient even under the low-UV conditions of Iceland because it occurs at long wavelengths.

For SO_2_, while scanning DOAS is useful, retrievals are associated with high errors and will struggle with very high UV absorption for emissions from some of the Icelandic melts, such as that fuelling the Holuhraun eruption. This presents a processing challenge, particularly as use of the longer wavelength regions of the spectrum can result in high errors that overestimate fluxes. Furthermore, very small differences in wind speed can produce huge variations in flux calculations—ideally, gas ratios should be used for accuracy in understanding volcanic gas emissions, especially where gusting or high winds occur.

In January 2015, we trialled passive FTIR traverses at the Holuhraun site and were able to use this method to detect sulphur dioxide. By this point in the eruption, magma supply was much lower than it had been in September, and the method was limited by a weak plume that was much dispersed. However, this method may prove significant in future eruptions in Iceland, particularly in the dark winter months.

During the 2021 and 2022 eruptions at Fagradalsfjall, experience from the earlier eruptions informed eruption management, even though the eruption rate was much lower than that of Holuhraun. IMO enhanced its collection of precipitation samples around the eruption site, and the measurement of lava field degassing by FTIR (Barsotti et al. 2023). In the early days of the eruption, there were also warnings about allowing animals to drink water close to the vents, due to the potential for pollution.

## Conclusions

We have presented multidisciplinary data concerning degassing at two very different Icelandic fissure eruptions. We find that such eruptions typically exhibit intermittent periodicity in their magma supply and/or gas supply. We have also presented BrO/SO_2_ data from both volcanoes, demonstrating that Icelandic magmas contain enough HBr to produce low but measurable levels of BrO by photochemical multiphase reaction cycles (“bromine explosion”), and that the levels of BrO relative to SO_2_ in the plume, after this initial rapid increase, then decrease with distance from the volcano. Modelling of the plume chemistry explains this trend through partitioning of BrO into other reactive bromine forms. We also presented ground-based traverse measurements of the SO_2_ flux during the summit eruption of Eyjafjallajökull.

Iceland presents particular challenges and opportunities for the monitoring of volcanic gases. There are large errors on UV spectroscopic retrievals in particular, due to the relatively low levels of UV at the latitude of Iceland, even around the equinoxes (when both of these eruptions took place). However, these issues can be mitigated to some extent through the use of powerful spectrometers, different fitting windows and co-addition of spectra. An active source could also be considered in future cases. In addition, FTIR can provide useful comparative data for DOAS measurements under these conditions.

Icelandic eruptions produce a very wide range of volcanic hazards. We analysed the degassing of the lava flows and showed that, in large magnitude basaltic eruptions, the near-field hazards derive largely from SO_2_ and potentially halogen emissions from the lava flows. This is consistent with historical reports concerning the haze from the Laki eruption of 1783-4.

## Supplementary Information

Below is the link to the electronic supplementary material.

Supplementary file1 (DOCX 6012 kb)

Supplementary file2 (WMV 27200 kb)

Supplementary file3 (XLSX 105 kb)

## Data Availability

Supplementary material contains additional datasets for glass and groundmass at Holuhraun and Fimmvörðuháls, and snow and particle size analysis for Fimmvörðuháls, as well as additional figures and a video. Raw spectra from UV-spectroscopic measurements can be provided on request in.std format. The raw FTIR data measured over the lava flow, using the spatter flow as a source, can also be provided on request in.sb format. Acoustic, thermal and video data can be provided in multiple formats.
