# Supplementary material for: Insights into volcanic hazards and plume chemistry from multi-parameter observations: the eruptions of Fimmvörðuháls and Eyjafjallajökull (2010) and Holuhraun (2014–2015)
Source: Nat Hazards (Dordr). 2023 Aug 19;119(1):463–95. doi: 10.1007/s11069-023-06114-7 (PMC10499761; doi:10.1007/s11069-023-06114-7)
Supplement: Supplementary file 1 — Supplementary file1 (DOCX 6012 kb) [file 11069_2023_6114_MOESM1_ESM.docx]

Appendix: Supplementary Figures

1. Thermal camera (Fimmvörðuhálsi)
2. Tephra particle sizes and Snow chemistry (Fimmvörðuhálsi)
3. Plots of DOAS data and spectrograms from acoustic data (Fimmvörðuhálsi)
4. Plots of FTIR data (Holuhraun)
5. Traverse map for summit eruption
6. Petrography of Holuhraun lava (SEM)

## A1: Thermal imaging

Thermal images were taken with a FLIR^TM^ P25 Thermal Camera (Forward-Looking InfraRed). Images in Figure A1 have not been corrected for temperature. Video AV1 has been corrected using a source emissivity of ε = 1. Images in the video were taken every 2 minutes.


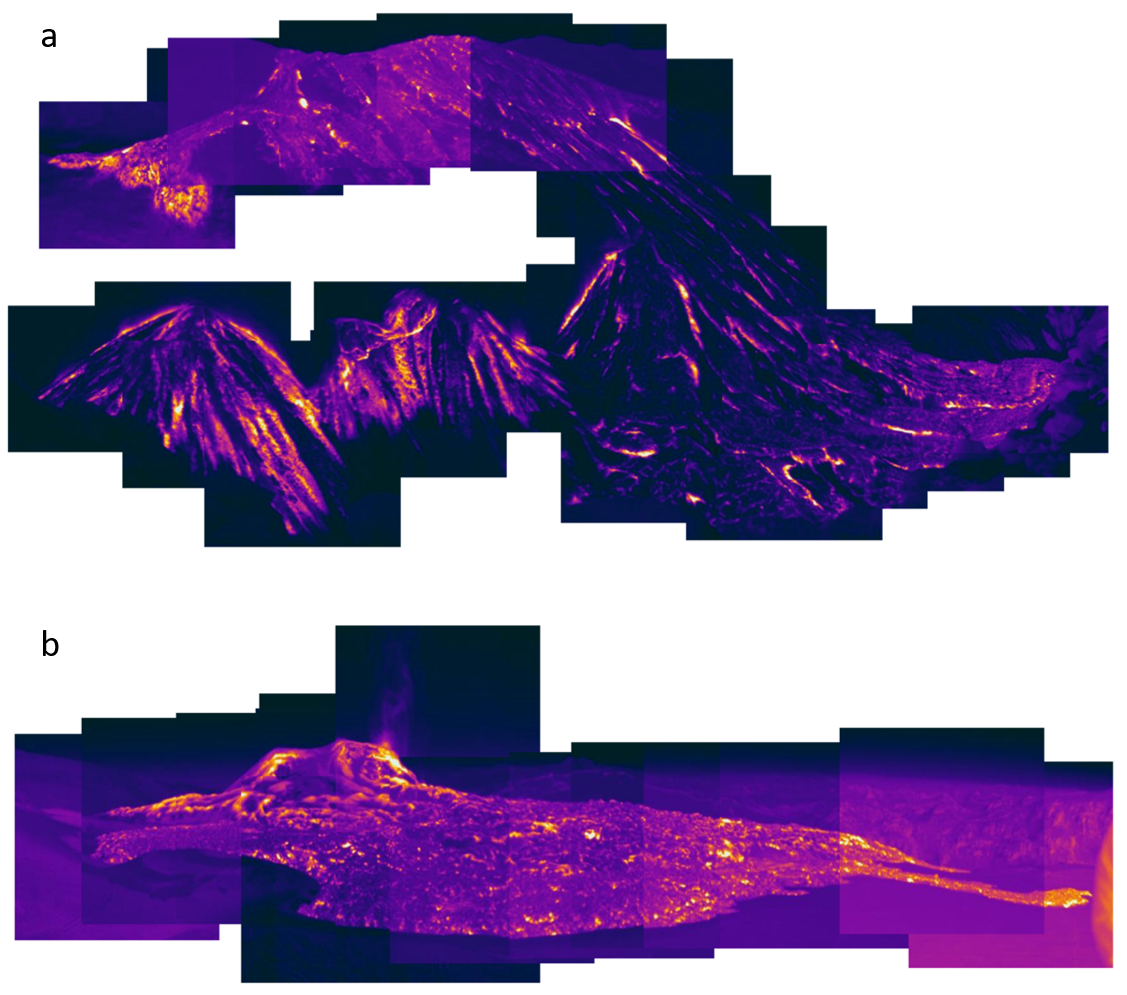


***Figure A1. Thermal camera composites showing (a) the “lava falls” into Hrunagil canyon (7 April 2010) and (b) the flow field at Fimmvörðuháls from Site B (7 April 2010). Note the narrow channels that form on the steeper slopes in (a).***

## A2: Particle size analysis and snow chemistry

Tephra was sampled at sites around the fissures at Fimmvörðuháls, where it was a few cm thick, on 7 April 2010. The samples were sieved for larger particles, and then small particle sizes were analysed using a Malvern Mastersizer. Three measurements were taken per sample to ensure consistency.

Snow was sampled at Fimmvörðuhálsi at a range of locations around the fissures and contained in clean plastic test tubes. A “neutral” sample was also taken at the edge of Myrdalsjokull for comparison. The snow samples were measured for Cr, Fe, Hg, K, Mg, Mn, Ni, Pb, S, Sr, V, Al and Ca using ICP-OES at the University of Cambridge. Snow and particle size data are provided in supplementary tables.


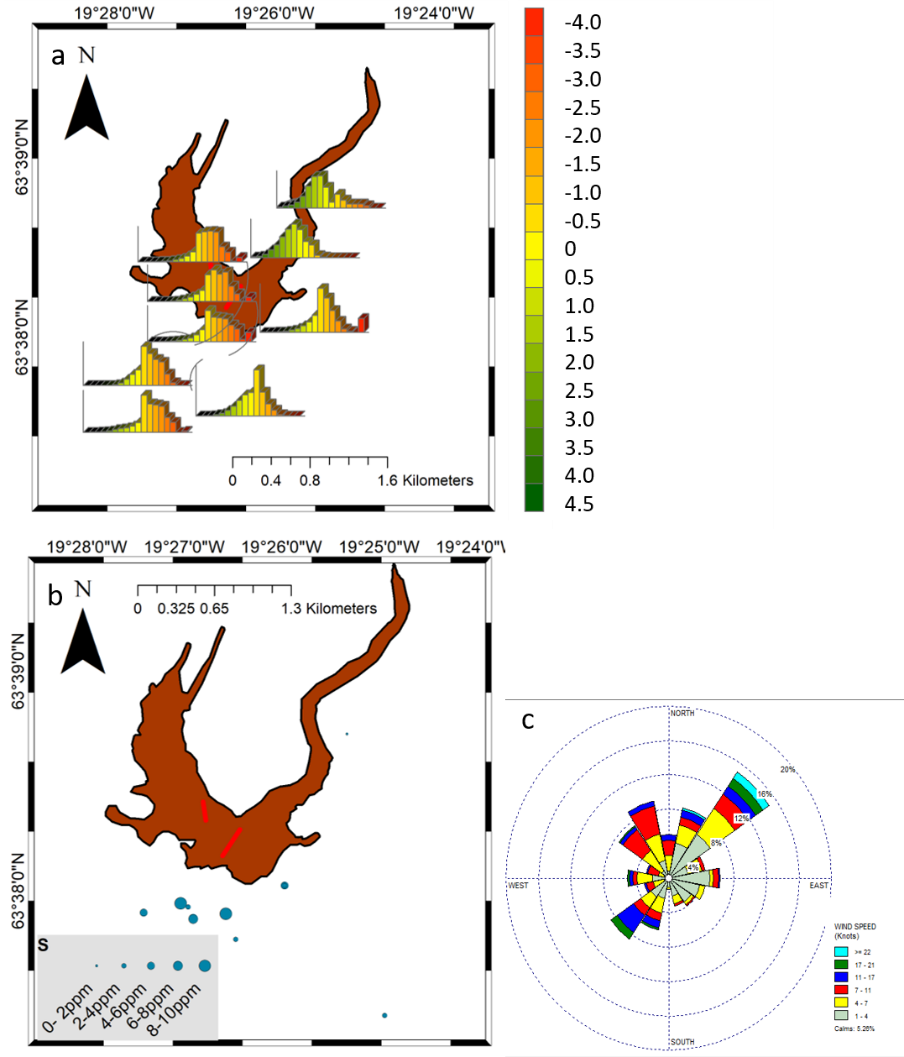


***Figure A2 (a) particle-size distributions of tephra samples collected on 4^th^ April at accessible sites around the fissure. Measurement scale is in phi units. (b) Sulphur content of snow samples collected at the same time but away from the tephra sampling sites. (c) Wind rose showing the pattern of winds over the site during the eruption up to 4^th^ April (data from Icelandic Met Office).***

## A3: Qualitative relationship between acoustic and gas datasets


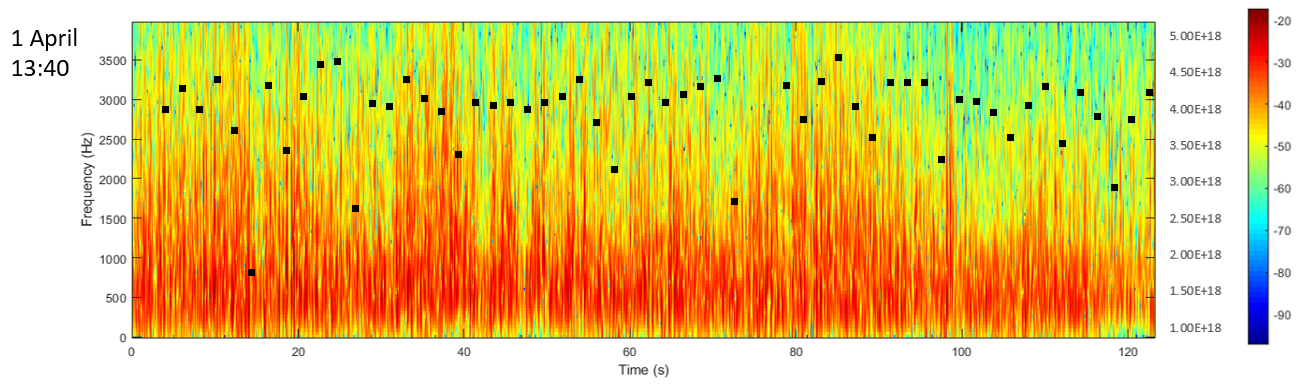

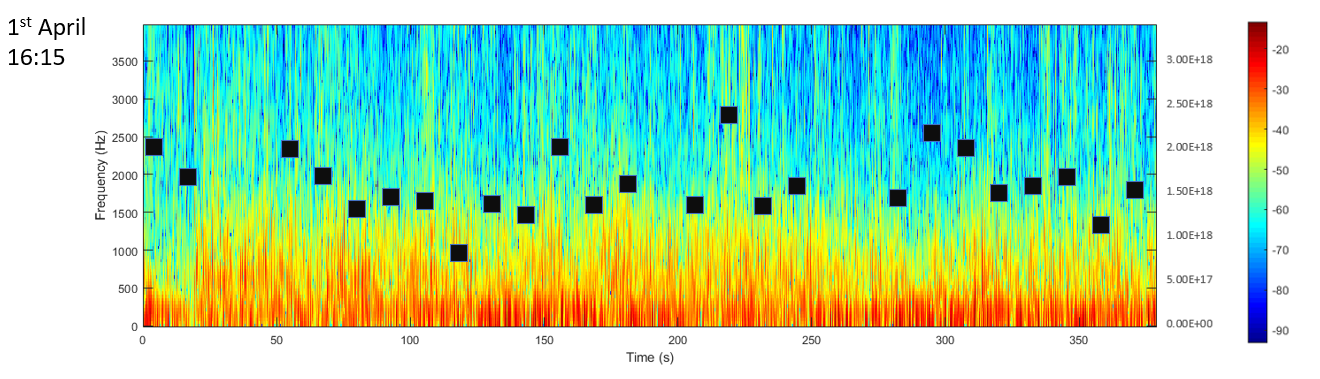


Note that this is a short time period of high activity in the middle of the day. SO2 is much higher here than later, when both signals are depressed:

***
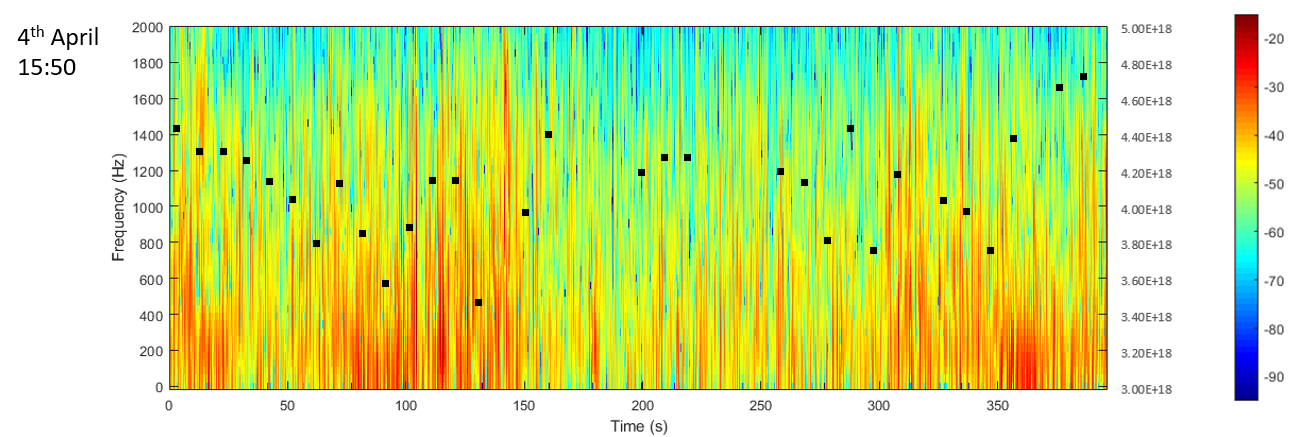
Figure A3a. Qualitative relationships between SO_2_ timeseries and acoustic data from 1^st^ April. The X axis on the periodograms applies to the DOAS data too. Estimated age of plume 10 sec. SO_2_ is in molecules/cm^2^. Colourbar shows db.***

***Figure A3b. Qualitative relationship between SO2 timeseries and acoustic data on 4^th^ April, this time at plume age 20 sec. SO_2_ is in molecules/cm^2^. Colourbar shows db.***

## A4: FTIR data from lava flow field (using flow on spatter cone as a source)

FTIR data were collected using the methods described in Pfeffer et al., 2017.

***Figure A4. OP FTIR data from the lava field.***

## A5 Traverses at summit eruption of Eyjafjallajökull


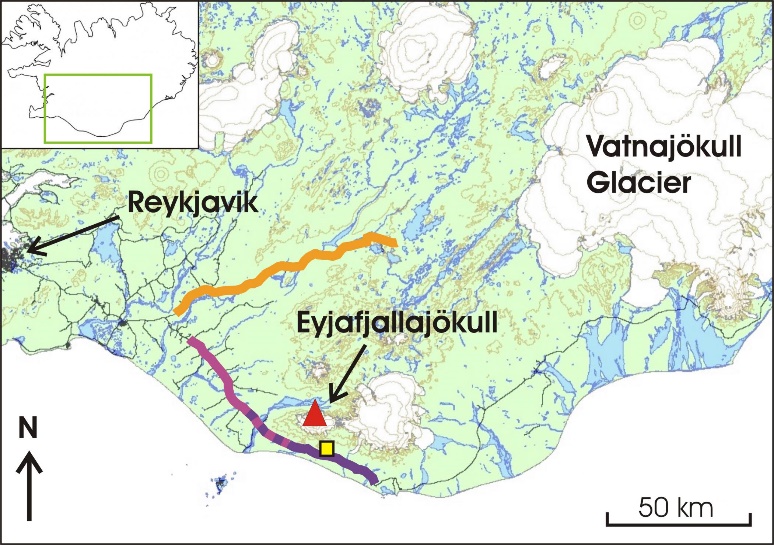


***Figure A5. Summit eruption. Plume traverse routes are marked in orange for 23 April (TR1 to 5), purple for 24 April in the morning (TR6-–7), and pink for 24 April in the afternoon (TR8-–10). Our location for stationary measurements on 24 April is represented by a yellow square.***

## A6: Lava texture at Holuhraun

***

***

***Figure A6. Lava sample taken ~7km from the vent, showing small pools of glass full of dendritic oxides.***
